# AI-based adaptive personalized content presentation and exercises navigation for an effective and engaging E-learning platform

**DOI:** 10.1007/s11042-022-13076-8

**Published:** 2022-06-29

**Authors:** Wafaa S. Sayed, Ahmed M. Noeman, Abdelrahman Abdellatif, Moemen Abdelrazek, Mostafa G. Badawy, Ahmed Hamed, Samah El-Tantawy

**Affiliations:** 1 Engineering Mathematics and Physics Department, Faculty of Engineering, Cairo University, Giza, 12613 Egypt; 2 Valeo, Egypt; 3 Mechanical Power Engineering Department, Faculty of Engineering, Cairo University, Giza, 12613 Egypt; 4 Computer Engineering Department, Faculty of Engineering, Cairo University, Giza, 12613 Egypt

**Keywords:** Dynamic learner model, Gamification, Learning style, Primary school mathematics, Reinforcement learning

## Abstract

Effective and engaging E-learning becomes necessary in unusual conditions such as COVID-19 pandemic, especially for the early stages of K-12 education. This paper proposes an adaptive personalized E-learning platform with a novel combination of Visual/Aural/Read, Write/Kinesthetic (VARK) presentation or gamification and exercises difficulty scaffolding through skipping/hiding/ reattempting. Cognitive, behavior and affective adaptation means are included in developing a dynamic learner model, which detects and corrects each student’s learning style and cognitive level. As adaptation targets, the platform provides adaptive content presentation in two groups (VARK and gamification), adaptive exercises navigation and adaptive feedback. To achieve its goal, the platform utilizes a Deep Q-Network Reinforcement Learning (DQN-RL) and an online rule-based decision making implementation. The platform interfaces front-end dedicated website and back-end adaptation algorithms. An improvement in learning effectiveness is achieved comparing the post-test to the pre-test in a pilot experiment for grade 3 mathematics curriculum. Both groups witnessed academic performance and satisfaction level improvements, most importantly, for the students who started the experiment with a relatively low performance. VARK group witnessed a slightly more improvement and higher satisfaction level, since interactive activities and games in the kinesthetic presentation can provide engagement, while keeping other presentation styles available, when needed.

## Introduction

Education is a main factor in the development, economic advance and welfare of communities [20]. Early stages of K-12 education are particularly important in acquiring the learning skills that will last with the students for the rest of their lives [15]. Bloom’s one-to-one tutoring and learning for mastery theories [8, 9] have always been respected by the educational community. Yet, the impracticality of their application, in conventional classroom teaching and teachers-to-students ratio, has always been a limitation [9]. It was not until the era of Information Communication Technology (ICT) and Artificial Intelligence (AI) that these theories became promisingly applicable [10]. There is a growing trend towards strengthening the dependence on distant-learning; expanding and developing E-learning platforms and; most importantly, introducing adaptive personalized features based on the needs and learning pace of each student [48].

A wide variety of E-learning and tutoring platforms provide the same content to all learners [34]. Fewer platforms provide personalized recommendation and adaptation features [48]. Learning styles can be defined as a set of cognitive, emotional, characteristic and physiological factors that serve as relatively stable indicators of how a learner perceives, interacts with, and responds to the learning environment [30]. Cognitive level stands for the learner’s level of knowledge. Adaptive personalized e-learning research focused on adult learners and considered learning styles and cognitive level as adaptation means [7, 42, 50, 62]. Most studies considered only one adaptation means and a corresponding target with a rule-based adaptation strategy [62]. As the number of adaptation means and targets increase, AI is needed to mimic human decision making for such various situations [61].

Many studies recommended multisensory learning [13] and offering students a set of multimedia learning material to choose from [32]. An adaptive personalized learning environment, which is designed to regulate different modalities based on their cognitive/learning styles, would be even better [32]. For students’ learning styles, various models and questionnaires exist but they were designed and validated for adults [28]. Hence, their modification to fit younger people represents a challenge. Since VARK provides a questionnaire specifically designed and validated for young children [35, 60], it can be used to initiate this dimension of our learner model. Based on the multiple intelligences and sensory modalities, it is the most convenient questionnaire to describe children learning behaviors as percentages of the different modalities [40, 49, 58]. In addition, it is one of the most effective learning styles dimensions utilized in personalization and adaptation [1].

On the other hand, gamification: “the use of game design elements in a non-game context”, is now the new trend in learning even in traditional classroom context [17, 29, 41, 59]. In games, children are more eager to accept challenges, rules, tasks, and feedback. They are even eager to enhance their skills to open higher levels of the game. Such game elements foster the creation of an immersive experience leading to higher involvement and engagement. Digital Game-Based Learning (DGBL) in K-12 education highly influences student’s motivation, engagement, and learning outcomes [17, 29, 41]. In a case study of elementary school students, teachers experienced a 13% increase in students’ attendance in mathematics classes after implementing gamification techniques inside the traditional classroom [2].

For students’ knowledge level, Bloom’s taxonomy classifies educational learning objectives into levels of complexity and specificity: Remember, Understand, Apply, Analyze, Evaluate, and Create [33]. It has been the primary focus of most traditional education and is frequently used to structure curriculum learning objectives, assessments and activities. Exercises difficulty scaffolding was implemented in several e-learning research studies based on cognitive level [43]. Yet, few studies provided a clear objective for this adaptation and they used rule-based adaptation [43]. Automatic detection of the suitable exercises difficulty enhances engagement and learning efficiency compared to self-regulated methods [45].

AI and machine learning have found their way to many applications such as: Internet of Things (IoT) [65], Intelligent Transportation Systems (ITS) [16], solution of mathematics problems [3, 4] and education [48]. RL is a form of machine learning that maps situations to actions in order to maximize the long-term reward. It differs from supervised and unsupervised machine learning approaches in the presence of learning agents. The learning agent senses the environment (despite its uncertainty), chooses the action that will maximize the rewarding function and updates the state accordingly [55]. Consequently, RL represents a very appropriate setup for an adaptive personalized e-learning environment and the proposed platform. DQN-RL leverages advances in deep learning to learn policies from high dimensional sensory inputs. Specifically, it learns with raw data using convolutional neural networks, instead of low-dimensional features vectors.

This paper proposes an Adaptive Personalized Platform for an Effective and Advanced Learning (APPEAL) for school students. APPEAL makes use of the previous literature from different aspects and aspires to bridge the gaps between different disciplines and overcome their limitations. It utilizes a novel combination of adaptation means, targets, strategies and goals. As adaptation means for learner modeling, APPEAL includes cognitive (learning style); behavior (effort, support and performance) and affective (engagement) categories of the student personal traits. A dynamic learner model, which detects and corrects the learner model in terms of learning style and cognitive level, is developed. For personalization and adaptation, APPEAL utilizes a DQN RL-based AI implementation and an online rule-based decision making based on student interactions as the adaptation strategy. It aims at achieving a personalized learning experience covering two main adaptation targets. First, it provides adaptive presentation through VARK for one group and gamification for the other group. Second, it provides adaptive exercises navigation and feedback. Adaptive navigation is achieved through scaffolding of exercises difficulty in Bloom’s taxonomy by skipping/hiding/reattempting. In addition, adaptive feedback is achieved through hints, attempts, and feedback messages. APPEAL aims at achieving the following adaptation goals: more effective learning with reduced time spent and improved grades and satisfaction level. To the best of our knowledge, no previous research study compared the learning outcome of using a combination of VARK modalities and gamification. Besides presenting the novel platform, APPEAL, with adaptive personalized presentation, navigation and feedback and its simulation based validation, the following research questions are assessed for both VARK and gamification groups in a pilot experiment:

- RQ1: Does APPEAL help improving aggregated-level academic performance and learning effectiveness indicators (pre- and post-test scores, completion time and learning efficiency) and how much improvement is achieved?
- RQ2: Does APPEAL help improving the data dispersion for these academic performance and learning effectiveness indicators and how much improvement is achieved?
- RQ3: Does APPEAL help improving disaggregated-level academic performance and learning effectiveness indicators for each student on lesson and exercises level?
- RQ4: Does APPEAL achieve good student engagement and satisfaction indicators?

The rest of the paper is organized as follows. Section 2 reviews some related researches on adaptive and personalized e-learning including e-learning systems for school students, gamified content and RL-based implementations. Section 3 demonstrates the adaptive personalized user interface provided by the front-end of APPEAL for both VARK and gamification groups displaying several screenshots. Section 4 is more concerned with the back-end architecture and adaptation algorithms: adaptive personalized RL-based VARK presentation and online rule-based exercises navigation. Section 5 presents and discusses the learning effectiveness results for the two experimental groups (VARK and gamification) and provides answers to the research questions of Section 1. Section 6 concludes the work and suggests future research directions. Finally, a list of all used symbols is given in the Appendix.

## Literature review

In many countries, ICT application is still less common in K-12 education compared to college and adult education [66]. Focusing on student modeling and corresponding adaptation for school students, several relevant studies in the last decade were reviewed. Table 1 provides a summary of these papers focusing on the following dimensions: purpose, design/methodology/approach and findings/implications/ recommendations. Although [21, 63] do not focus on dynamically detecting nor correcting the learner model; they show how effective the teaching style is in the academic performance of the learners for school students. Study [47] cluster/detect learner model in order to assess the preferable adaptation strategies without implementing any adaptation. Studies [39, 56] showed the importance of learning style correction through a dynamic learner model; however, the corresponding adaptation strategies were not investigated. Studies [11, 18] focus on the content adaptation based on static learner model for cognitive level detection. Although [14] considered dynamic changes of the model with the interactions, this system did not consider the learning style. Study [12] proposed a model-tracing intelligent tutor that interpret students’ mathematical problem-solving behaviors in fractions topic and provide adaptive personalized lower level exercises and feedback. Study [25] provided adaptive exercises navigation (skipping) and feedback (hints), which resulted in scores improvement from pre- to post-test. Study [24] discussed adaptive e-learning focusing only on exercises omission for high performing students. Yet, the system [24] did not adapt the learning content according to any learning style model. Other studies such as [19] used a unified presentation, as opposed to personalized, but in the form of mathematics worked example videos. Yet, it led to better results than the traditional method and a students’ survey revealed their satisfaction.

**Table 1:** Summary of the reviewed papers targeted at school students

Ref.	Purpose	Design/methodology/approach	Findings/implications/recommendations
[21]	Personalize teaching style	Questionnaire (active/reflective learning styles)	Enhanced post-test scores
[63]	Personalize assessment and annotation	Pre-test (knowledge level)	Enhanced post-test scores
[47]	Cluster learners	Learner knowledge tracing	Only recommendations to teachers about the role of adaptation
[39]	Detect the preferred navigation pattern	Questionnaire (MMTIC learning styles)	Dynamic model matches questionnaire
	- Detect dynamic learner model	Interactions (navigation)	
[56]	Detect the preferred learning pattern	Questionnaire (Jackson’s learning styles)	- Only recommend the most chosen pattern and resulting in higher grades, which does not belong to the area of personalization
[11]	Adapt content	- Pre-test (knowledge level)	- Enhanced post-test scores
		- Questionnaire (motivation)	- Enhanced motivational scale
[18]	- Allow hints and attempts upon request	- Pre-test (knowledge level)	Enhanced post-test scores
	- Adapt feedback	- Interactions (Correct/wrong answers and requests)	
[14]	- Detect and correct dynamic learner model	Interactions (knowledge level)	Enhanced test scores than control group
	- Adapt content		
[12]	Adapt feedback	Automatic mistakes detection (lexical, syntax and semantic analyzers)	Enhanced post-test scores and compared to control group
[25]	- Adapt exercises navigation	Expert knowledge structure	Enhanced post-test scores
	- Adapt feedback		
[24]	Adapt exercises navigation	Interactions (score, time, effectiveness)	- Enhanced time
			- Conserved score and increased effectiveness
APPEAL	- Detect and correct dynamic learner model	-Interactions (score, time, hints, attempts)	Answers to RQ1-4 in Section 5
	- Adapt presentation, exercises navigation and feedback	- DQN-RL	- Enhanced post-test score, learning gain, time, effectiveness and satisfaction
	- Compare adaptive personalized VARK presentation to gamification	- Online rule-based decision	- Enhanced post-scores for all students and reaching the most advanced level - Slightly better results for VARK group over gamification

In summary, the reviewed relevant works focusing on school students suffer from the following general limitations:

- Most of them were based on static learner models through questionnaires and even did not proceed to implement an adaptation module or provide adaptation rules.
- Most of them did not cover all elements of the learning process or branches of adaptation of content presentation and exercises navigation.
- Some of them did not focus on the implementation technique of the learner or adaptation models and most of them did not employ AI-based solutions.
- Most of them did not provide clear evidence or enough performance metrics of the learning effectiveness of their proposed systems.
- Most of them did not consider the affective state of the student, engagement or satisfaction surveys.

As for gamification, interactive games yielded an improvement in post-test performance in English teaching in [44], either depending on computer mouse or motion sensing. After-school remedial instruction of mathematics was considered in [37], where the game-based group outperformed the video grou.p although both resulted in good performance. The effect of gender on the verbal and nonverbal engagement behavior in game-based learning was explored in [22], which observed that male students were more engaged.

From the viewpoint of adaptive and personalized e-learning implementation, RL is proven to be an effective approach [5, 6, 26, 27, 36, 51, 52, 57]. Yet, these works also suffer from the following limitations:

- Objectives of the studies focused on either learning style detection or academic level improvement. However, there is a lack of studies that consider both dimensions.
- On the level of academic performance improvement, the reward function, which directs RL towards the most suitable action, usually focused on either improving the students’ performance (grades) or reducing their effort (time on task) but not both.
- These studies mostly targeted college level and adult learners. RL was utilized for elementary school students’ learning assistance in computer skills in [23]. Yet, the only provided adaptation target was adaptive personalized feedback with the goal of saving teachers’ time.
- Deep RL has not yet been widely employed in this field although it shows promising results [53]. The authors of [53] aimed at improving students’ learning performance and reducing their time on task, but not simultaneously. In addition, the adaptation was limited to deciding between worked examples or problem solving.

APPEAL attempts to overcome these limitations by developing an adaptive personalized e-learning platform targeted at school students with a DQN-RL-based implementation. A dynamic learner model is developed and, accordingly, content presentation, exercises navigation and feedback are adapted altogether. APPEAL’s goal is to improve the students’ learning performance, reduce their time on task and achieve a high satisfaction level. To asses the achievement of this goal, various aggregated-level, statistical and disaggregated-level academic performance, learning effectiveness and satisfaction level indicators are investigated. To the best of our knowledge, no previous research study compared the learning outcome of using a combination of VARK modalities and gamification.

## APPEAL user interface

**Fig. 1:**
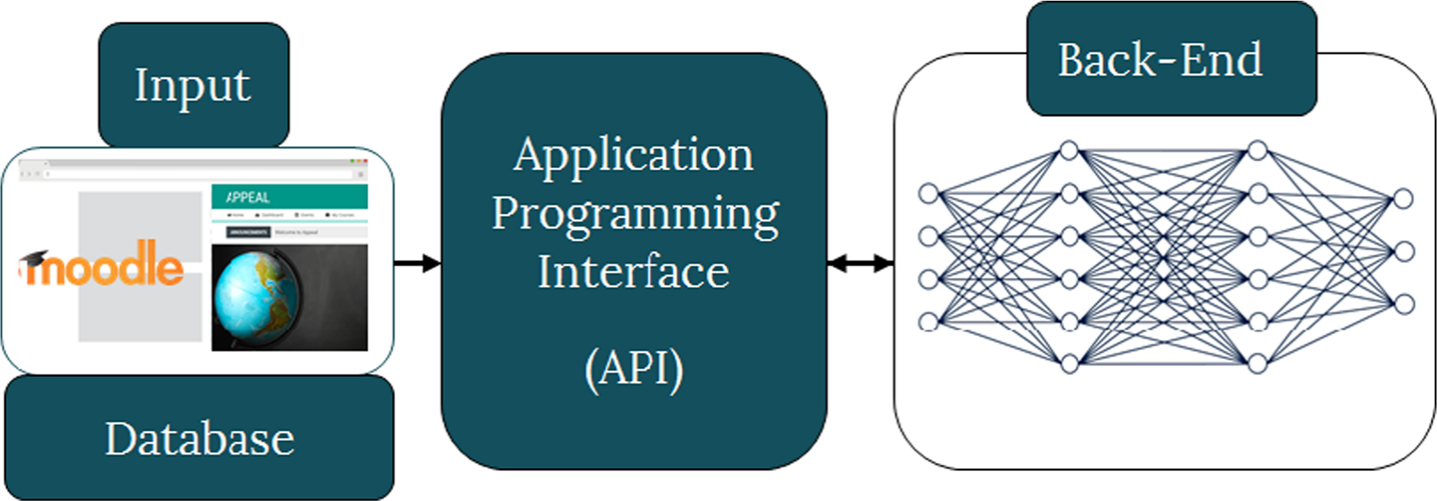
APPEAL general architecture

Figure 1 shows the general architecture of APPEAL, which is built as a dedicated website based on Moodle. As an adaptive personalized e-learning platform, APPEAL consists of the front-end, back-end, and the Application Programming Interface (API) between them. The front-end includes all user interface and it is where all student interactions take place. The back-end includes all the rule-based and machine intelligence-based algorithms that make all the decisions, i.e., what to present and when for each student based on his/her interactions. The API is the interface through which the first two components: front-end and back-end communicate. Details of the three components of APPEAL are given in this section and the next section.

**Fig. 2:**
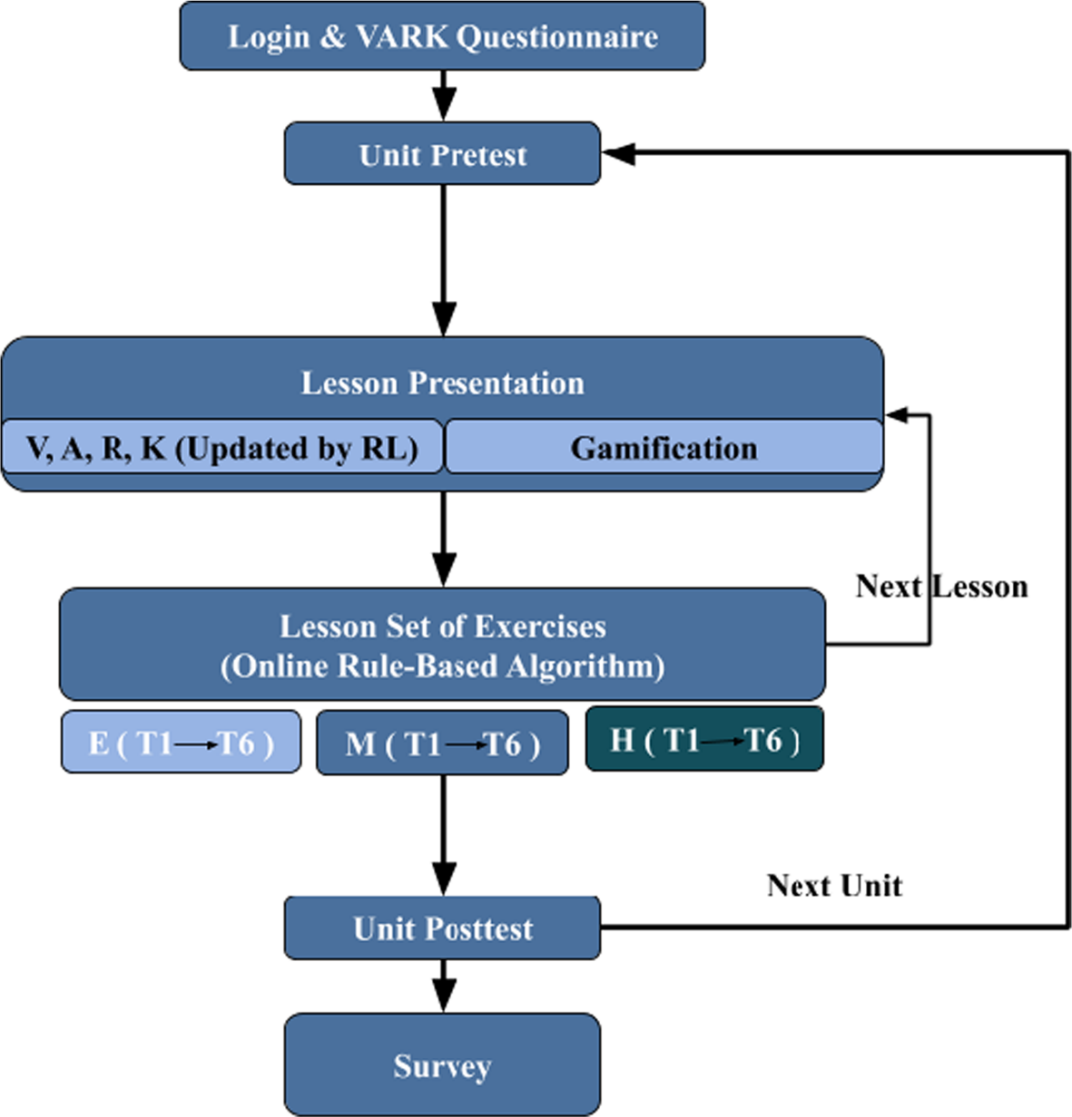
Flowchart of the student’s journey through APPEAL

The student’s journey through APPEAL is shown in Fig. 2. It starts by logging into his/her account. The student takes an initial VARK questionnaire and proceeds to the lesson presentation based on his/her group (VARK versus gamification). For each unit of the curriculum, the student takes a pre-test to determine his/her entry level. The lessons are presented into different presentation styles/modalities or gamified including explanation and examples. After each lesson, there is a set of exercises that cover Bloom’s taxonomy 6 categories (T1 to T6). Each taxonomy category is further measured by three levels of exercise difficulty: Easy-Medium-Hard (E-M-H). Only a subset of each taxonomy’s exercises is displayed to the student based on his/her level. Both the easy and medium levels with lighter colors are subject to hiding/skipping if the student interactions reflect an advanced level. Reattempting some levels with equivalent exercises from the question bank can also take place when needed till mastery. The aim is to improve student level such that he/she becomes capable of solving hard exercises despite his/her entry level. Throughout these steps, the student interactions: time spent, attempts, hints and grades are recorded. In addition, they are communicated to the back-end adaptation algorithm to modify its decisions accordingly. After each unit, the student takes a post-test to determine his/her exit level. Finally, students are asked to fill out a satisfaction-feedback survey.

**Fig. 3:**
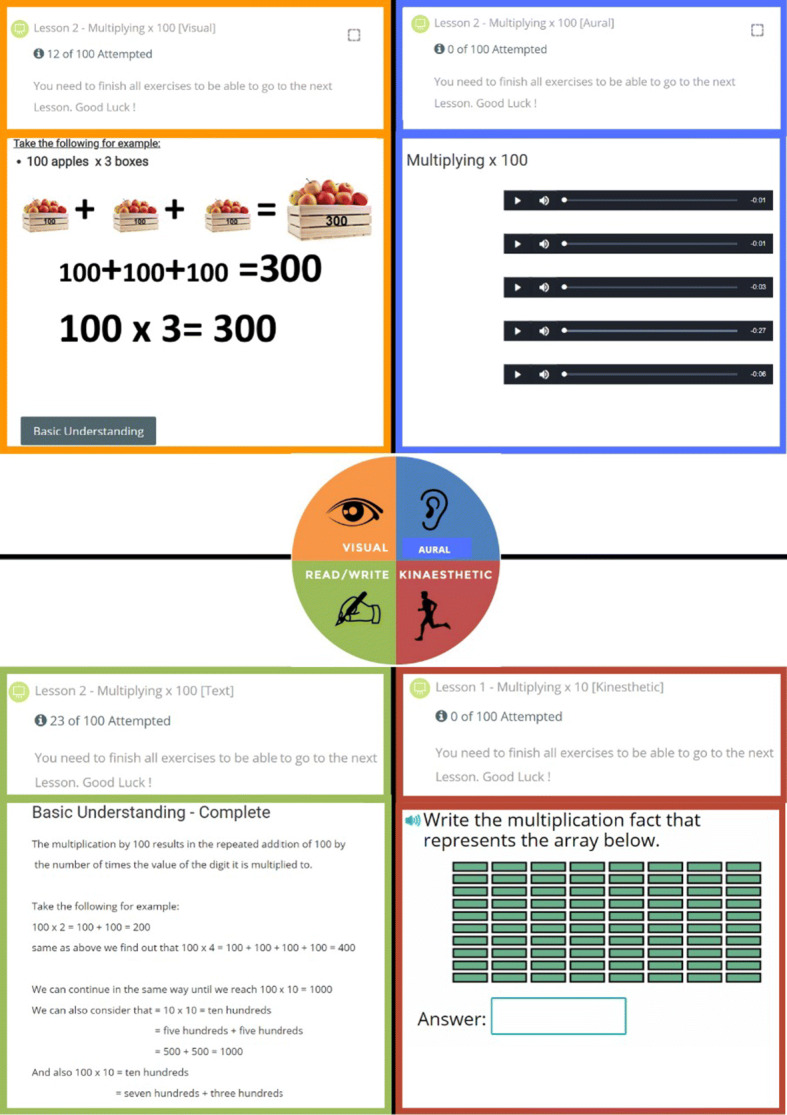
Four different lesson presentation modalities for VARK group

The front-end of the official website APPEAL is designed based on Moodle. The content and tests of Grade 3 mathematics was prepared under the supervision of school teachers. All lessons were prepared in the four presentation modes/styles: visual, aural, text and kinesthetic. Figure 3 shows samples from the four different lesson presentation modalities including explanation and examples for VARK group. The back-end algorithms (Section 4) determine which presentation will be given to the student in an adaptive manner.

**Fig. 4:**
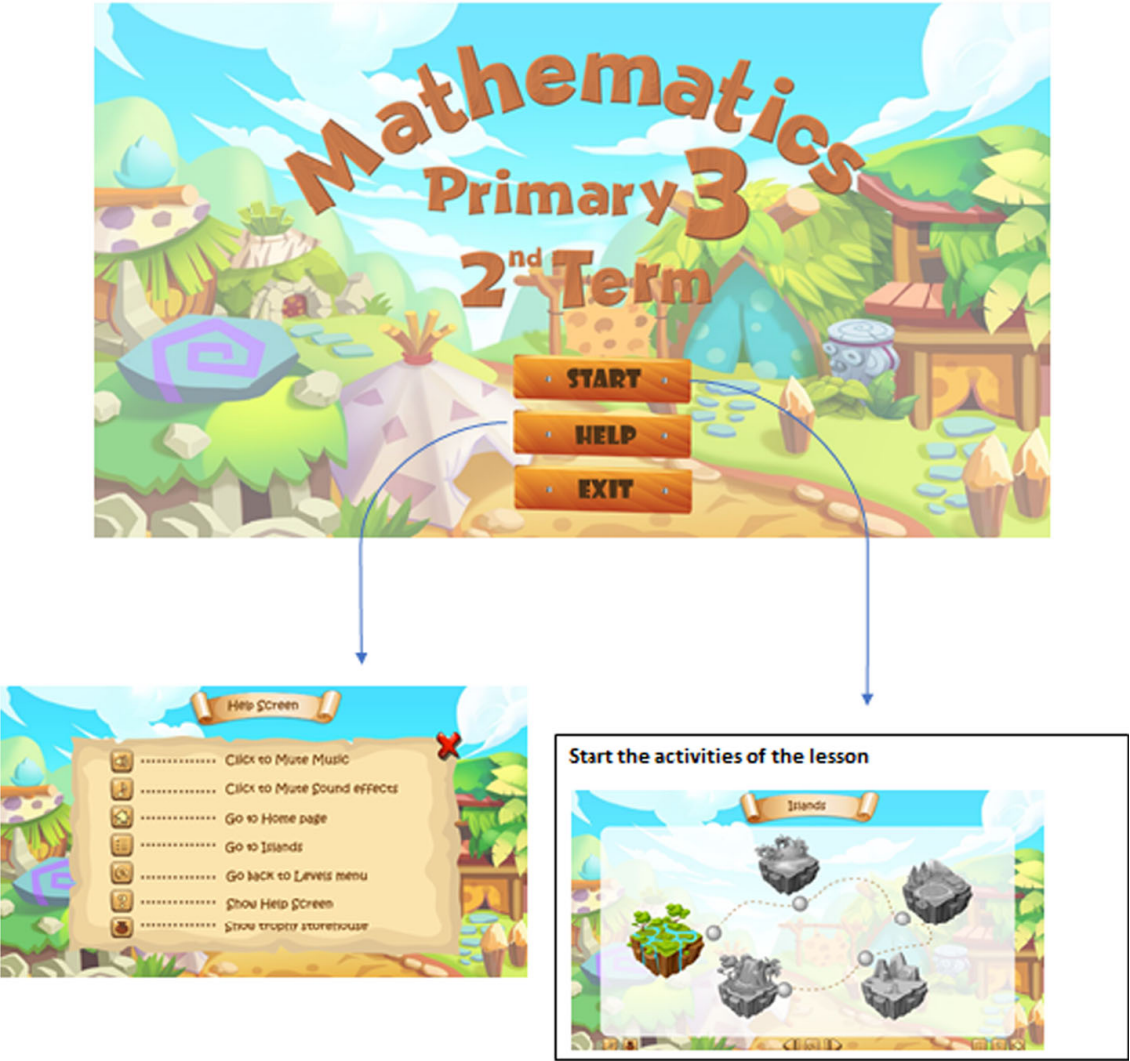
Home, start and help screens for gamification group

**Fig. 5:**
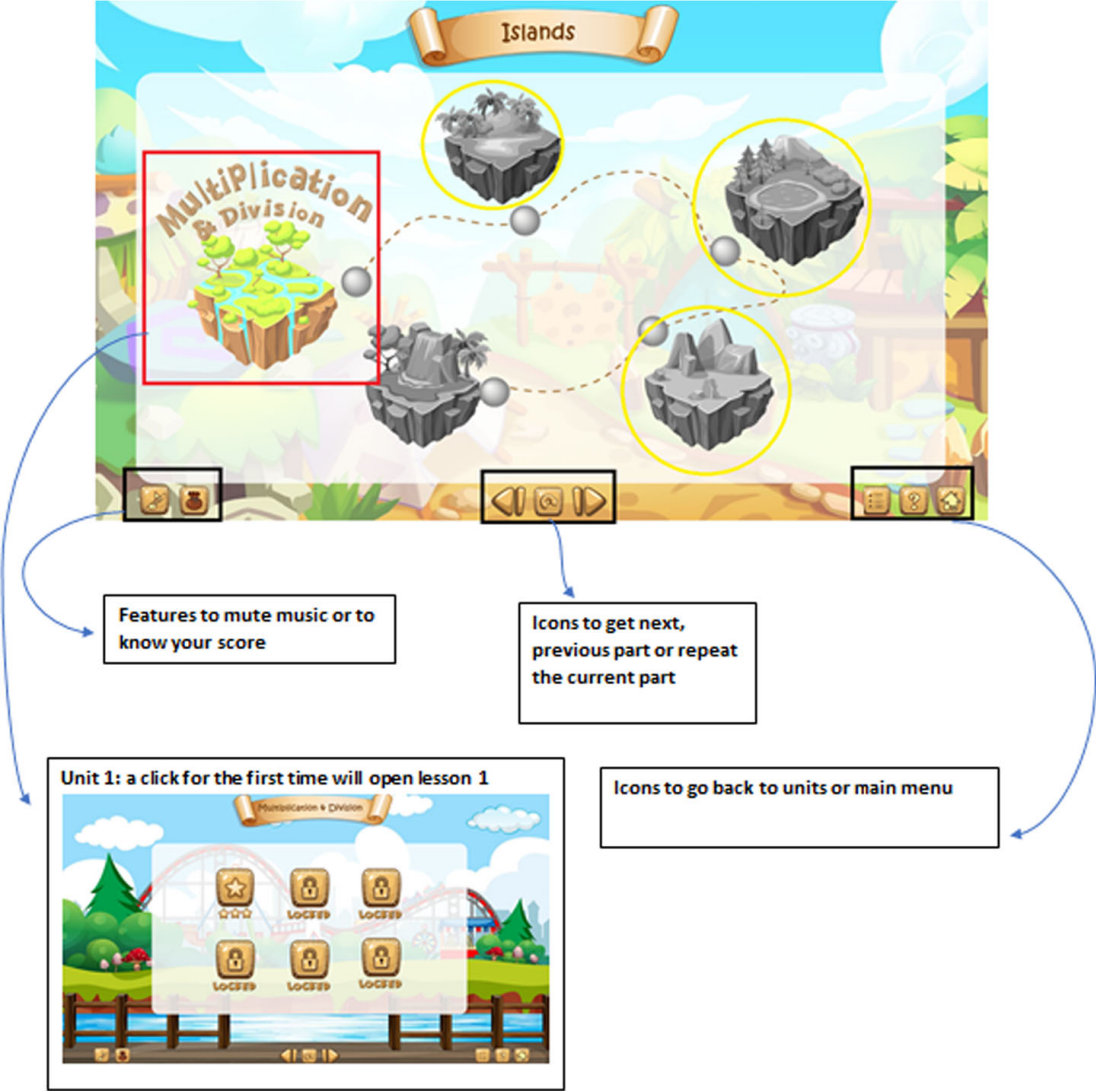
Units and lessons navigation and features for gamification group

**Fig. 6:**
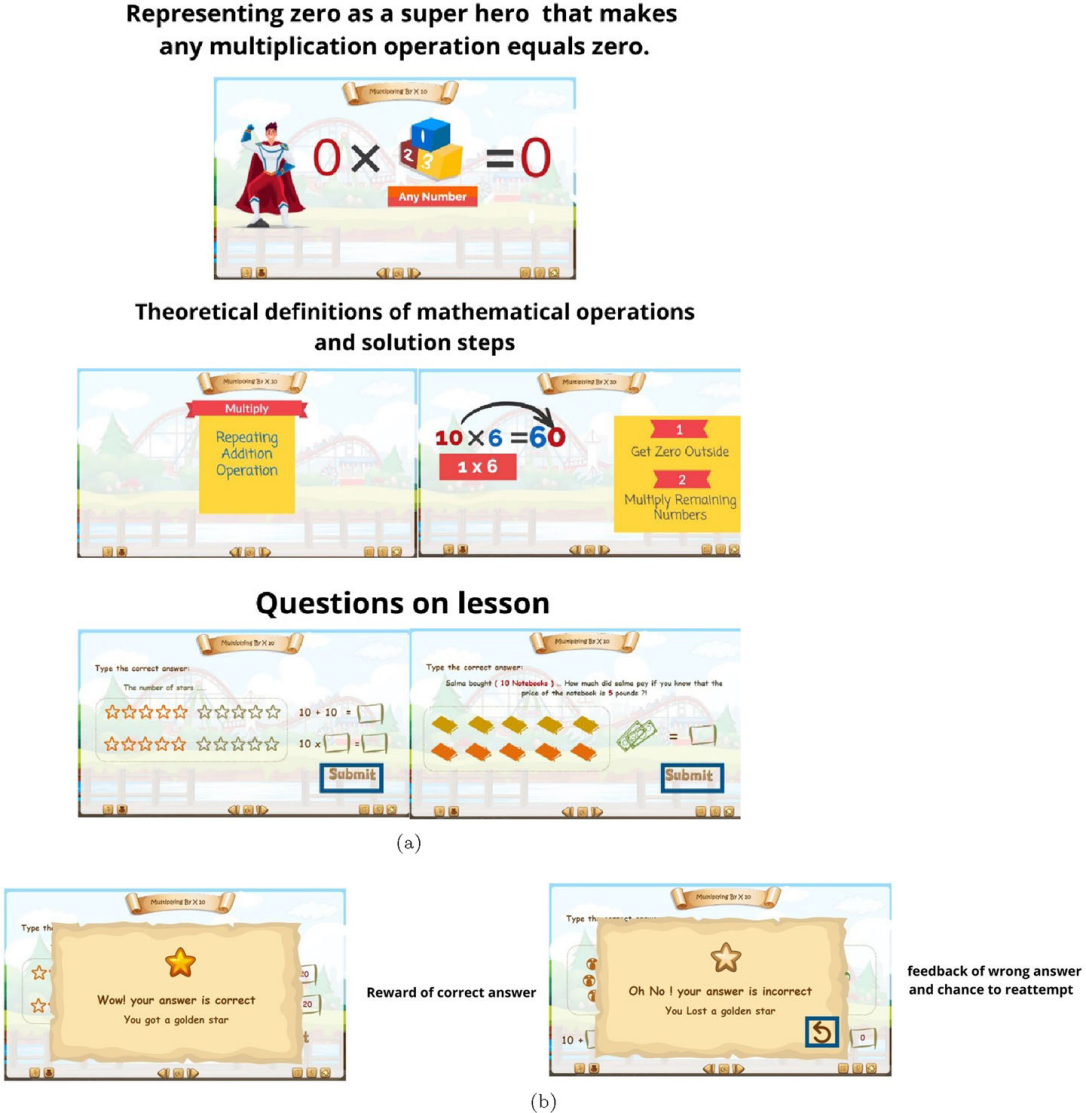
Lesson (a) explanation and (b) examples samples for gamification group

The gamification part of APPEAL focuses on the student affective state by targeting increased engagement. The design includes features like offering virtual markets, a pointing system, progress bar, and interactive quizzes in the form of challenges and competitions to enhance students’ motivation, eagerness to learn more and engagement. The lessons are prepared as Shareable Content Object Reference Model (SCORM) e-learning software products. Figure 4 shows the gamification interface including home screen with start, help and exit icons as well as the screens that appear upon clicking on these buttons. Figure 5 provides an example on the details of units and lessons navigation in a storytelling form, where the student is the main character navigating from an island to another. The function of each icon and the features provided by them are also shown including music, controls, replay, navigation, … etc. In addition, locked levels encourage the students to proceed in the learning process to unlock them and feedback is provided in the form of score follow-up in the form of a pointing system with progress bar. Figure 6 demonstrates the main features of explanation and examples with snapshots from the gamified scenario of a lesson.

**Fig. 7:**
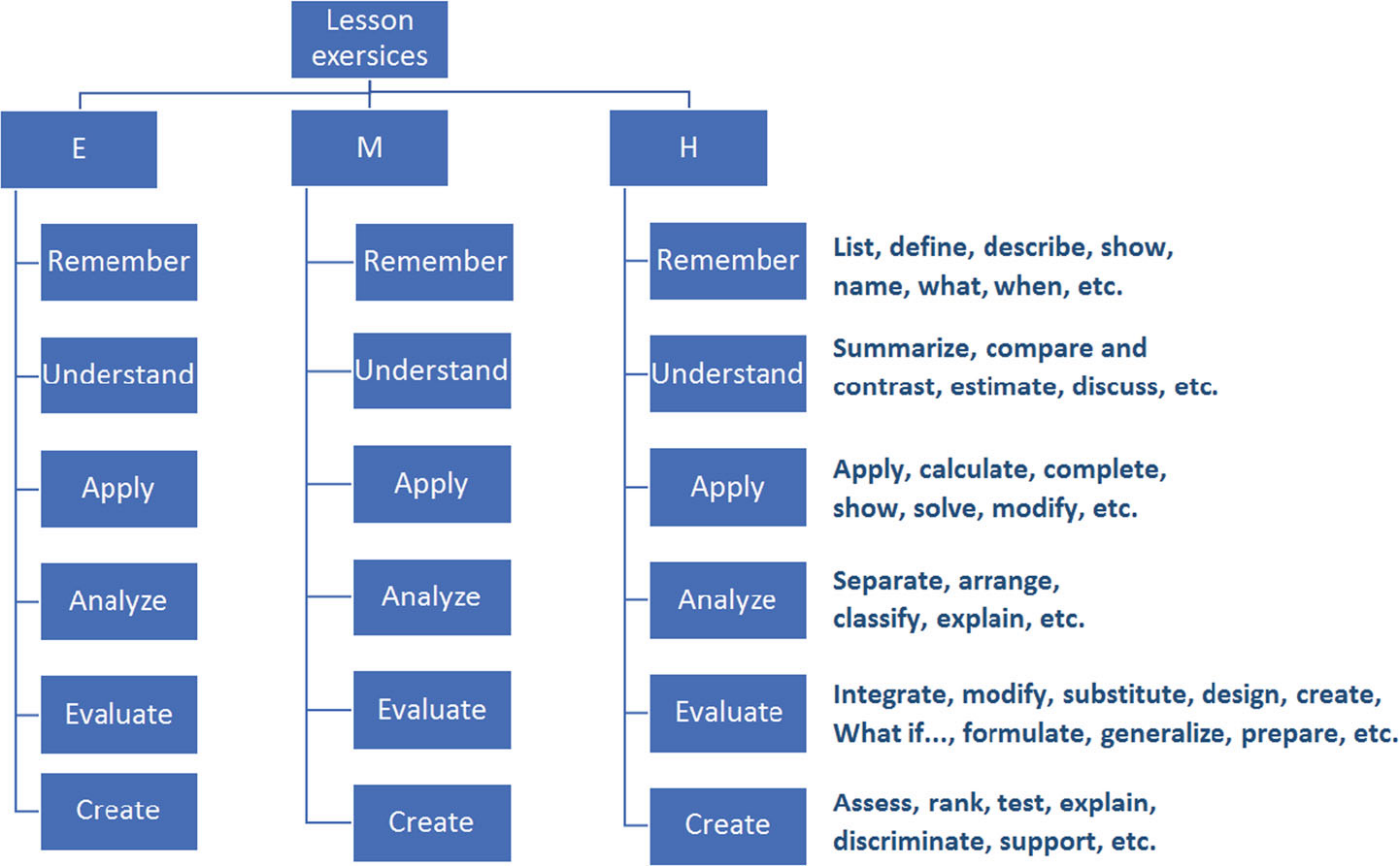
Structure of the exercises bank per lesson

**Fig. 8:**
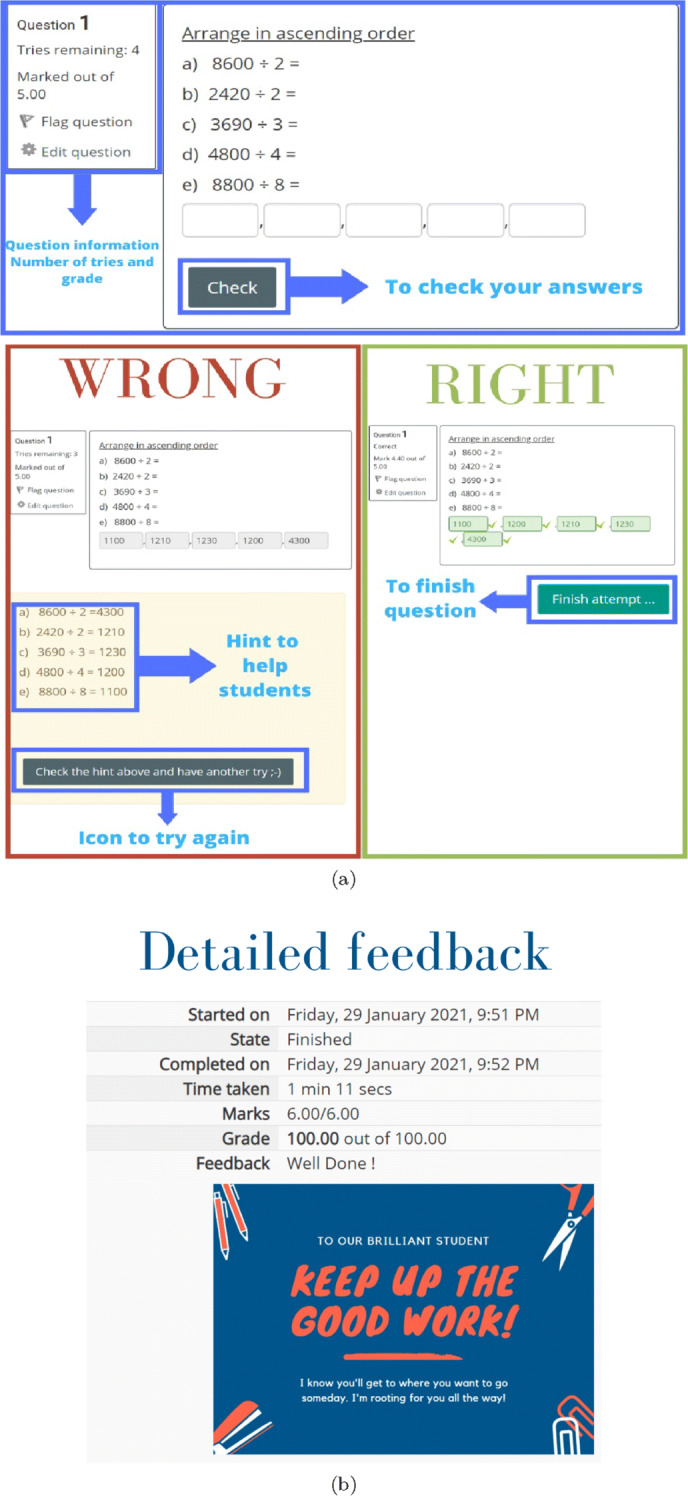
Sample screenshots from the (a) exercises and (b) feedback to the students in both groups

Exercises were provided and classified by the teachers based on Bloom’s taxonomy categories (T1 to T6) according to their objectives and (E-M-H) according to their difficulty level. The structure of the exercises bank per lesson is shown in Fig. 7, where 3 × 6 = 18 exercises cover the three difficulties for each taxonomy level. Teacher accounts can add content and exercises and access the student data of their classes. They add the exercises to the corresponding classification based on the nature of questions under each Bloom’s taxonomy level. The academic level of the learner among E, M and H is initialized by his/her level in the pre-test and updated from previous interactions. In addition, the back-end algorithms (Section 4) determine which difficulty level he/she will start at or navigate to. Figure 8 shows some screenshots from the exercises and the feedback given to the students at different situations, which are common for both groups.

## Adaptation algorithms

**Fig. 9:**
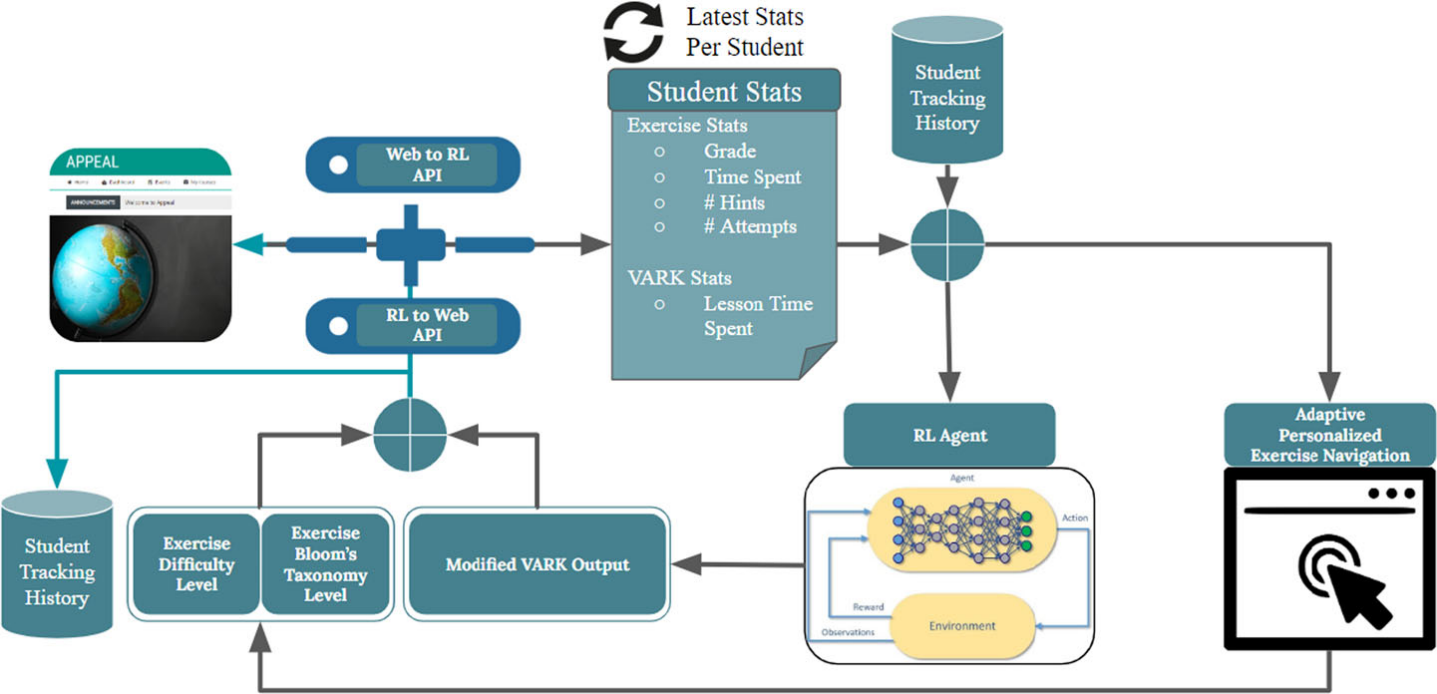
APPEAL back-end architecture and communication with front-end

The back-end of APPEAL receives the student state based on his/her interactions with the front-end, as well as the student tracking history, as shown in Fig. 9. Then, it adapts content presentation according to the student’s learning style using an RL algorithm. In addition, it adapts the exercises navigation based on his/her knowledge/academic level using an online rule-based decision making algorithm. We start by explaining the exercises navigation as the student state is mostly obtained from his/her performance in the exercises.

### Adaptive personalized exercises navigation and feedback

A unified online rule-based decision making algorithm based on student interactions is used. Depending on the student behavior: effort (attempts), support (hints) and performance (grades) as adaptation means, adaptive personalized exercises navigation aims at determining the appropriate exercises difficulty within each Bloom’s taxonomy level. The algorithm determines the difficulty level of the exercise that appear to the student at entry level per taxonomy category (*tax*), where *difficulty* ∈{1,2,3} corresponding to E,M,H, respectively. For simplicity, the difficulty is initialized by the pre-test score of each unit for all taxonomy categories as follows:

1 $$ {difficulty}_{Pre}= \begin{cases} 1, \quad & \qquad {Pre} < 50, \\ 2, \quad & \qquad 50 \leq {Pre} <75, \\ 3, \quad & \qquad 75 \leq {Pre}. \end{cases} $$

Throughout the student’s journey through APPEAL, the following algorithm keeps track of his/her level per taxonomy category based on a penalized grade, *grade*_*penalized*_, and decides the difficulty of the exercise to be displayed next. The decision or action corresponds to improvement/upgrading (+ ), no change (0), or deterioration/downgrading (−). The penalized grade implicitly includes the attempts and hints. *grade*_*penalized*_= 1 when the student gets the correct answer(s) by himself/herself. With every hint up to the available 4 attempts, *grade*_*penalized*_ is computed from grade by subtracting 0.2, where *grade* ∈ [0,1]. If the last attempt failed, the answer is displayed and *grade*_*penalized*_= 0. Two thresholds are defined as *th*_*g*1_= 0.4 and *th*_*g*2_= 0.8. Separate decisions are made for each taxonomy category as given in Algorithm 1.

Students with penalized grade less than 0.4 have not provided the correct answers and are downgraded to a lower level (− 1). Students with penalized grade between 0.4 (inclusive) and 0.8 (exclusive) have reached the correct answers by themselves after given two or three hints and can be upgraded to one level higher (+ 1). Students with penalized grade greater than or equal to 0.8 have reached the correct answers directly or after a single hint and deserve to be upgraded to two levels higher (+ 2). Correct answers of the hard exercises in all taxonomy categories are considered as a pre-requisite for the next lesson. Upon reaching the hard exercises in all taxonomies, the student must answer them to unlock the next lesson. If all hints were used and the correct answer is displayed to the student, unlocking the next lesson requires answering an exercise of the same level. This guarantees the aim of APPEAL that the student level improves to the level that corresponds to hard exercises in all six taxonomy categories. Then, next lesson difficulty is initialized from pre-test score, and so on. 
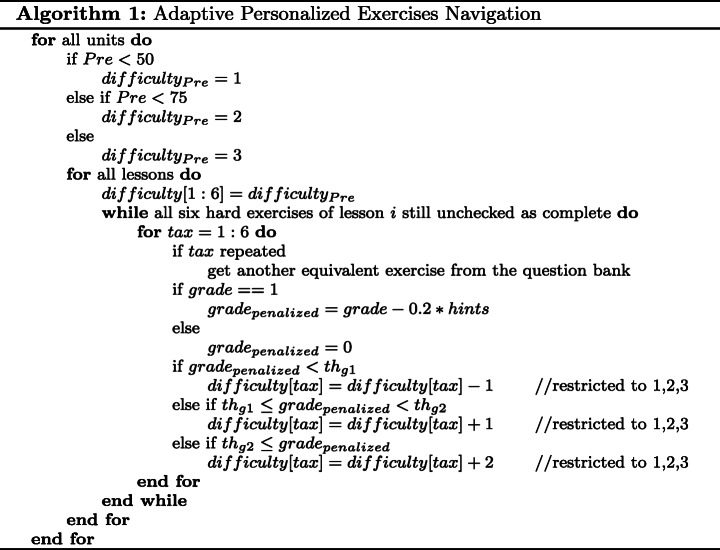


### Adaptive personalized VARK presentation using RL

Adaptive personalized presentation can be formulated as a stochastic control problem, more specifically Markov Decision Process (MDP), where the state of environment is represented by the interaction of the student with the E-learning platform. Such interactions are partially random and partially affected by the platform. They have stochastic features especially at an early age where the personality traits and character are still being developed. The MDP problem is optimally solved using dynamic programming and practically solved using function approximation or deep reinforcement learning due to the curse of modeling and curse of dimensionality issues [55]. MDP is defined by a tuple < *S*, *A*, *R*, *T*, *γ* >, which consists of a set of states *S*, a set of actions *A*, a reward function *R*(*s*, *a*), a transition function $T (s,a,s^{\prime })$ that equals the probability distribution $P(s^{\prime }|s,a)$, and a discount factor *γ*. In each state *s* ∈ *S*, the agent takes an action *a* ∈ *A*. Upon taking this action, the agent receives a reward *R*(*s*, *a*) and reaches a new state $s^{\prime }$, determined from $P(s^{\prime }|s,a)$.

A policy *π* specifies for each state which action the agent will take. The goal of the agent is to find the policy *π*, which maps states to actions, that maximizes the expected discounted total reward over the agent’s lifetime. The value *Q*_*π*_(*ϕ*(*s*),*a*) of a given state-action pair (*s*, *a*) is an estimate of the expected future reward that can be obtained from (*s*, *a*) when following policy *π*, where *ϕ*(*s*) = *s* in our case. The optimal value function *Q*^∗^(*s*, *a*) provides maximal values in all states and is determined by solving the Bellman equation:

2 $$ Q^{*}(s,a)=E\left[R(s,a)+\gamma \sum\limits_{s^{\prime}}P(s^{\prime}|s,a) {\max}_{a^{\prime}} Q^{*}(s^{\prime},a^{\prime}) \right]. $$

The optimal policy *π* is then *π*(*s*) = argmax_*a*∈*A*_*Q*^∗^(*s*, *a*).

#### State-action-reward < *s*, *a*, *r* > triplet

APPEAL provides personalized presentation using an RL approach based on both cognitive (learning style) and behavior (effort, support and performance) categories of the student personal traits. As previously explained, the learning agent of RL senses the current state from the environment, chooses the action that will maximize the rewarding function and updates the next state accordingly [55].

**Fig. 10:**
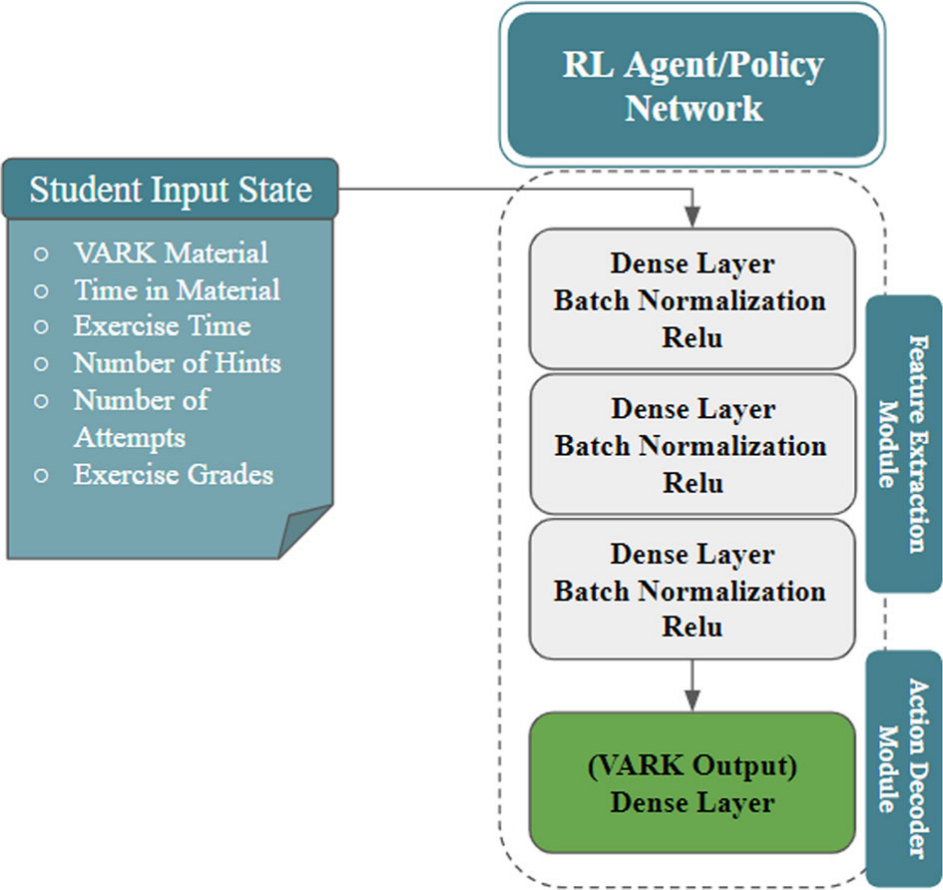
RL agent
- Action is defined by the adaptation module as the detected learning style (VARK) with the corresponding presentation to the lesson explanation material in the form of four binary digits, where only styles with “1” are presented while others with “0” are hidden.
- Reward is defined as the improvement in the academic performance of the student and the saving in the time spent, hints checked and number of attempts per lesson, which are supplied as the input state from the API.

- State is defined by the student interactions with the domain module, i.e., time spent per lesson (effort), scores per exercise (performance), number of hints checked (support), and number of attempts of the same taxonomy category and difficulty level (effort). Student state definition is also given in Figs. 9 and 10.

#### RL agent

The utilized algorithm is DQN with experience replay given by Algorithm 2 [38]. DQN approximates the value function *Q*(*s*, *a*) with a deep neural network that outputs a set of action values *Q*(*s*, *a*;*𝜃*) for a given state input *s* and action *a*, where *𝜃* are the parameters of the network. There are two key components of DQN that make this algorithm work. First, it uses a separate target network $\hat {Q}(s,\cdot ;{\theta }^{-})$ with separate weights *𝜃*^−^, which are copied every *C* steps from the regular network, so that the target *Q*-values are more stable. Second, the agent adds all of its experiences to a replay buffer *D*, which is then sampled uniformly to perform updates on the network. An experience is the tuple $<s,a,r,s^{\prime }>$.

An *𝜖*-greedy policy is used to generate actions as follows. Select the action that gives the maximum *Q*-value with probability (1 − *𝜖*) or select a random action with probability *𝜖*. The probability *𝜖* decreases through learning as the number of iterations increases, which is known as annealing. All the interactions of the user/student are stored in memory. The next action is determined by the maximum output of the Q-network *a* = argmax_*a*∈*A*_*Q*(*s*, *a*, *𝜃*) when using the optimal policy. The loss function is defined as follows after sampling a random minibatch of transitions $(s_{j},a_{j},r_{j},{s_{j}}^{\prime })$ from *D*:

3a $$ y_{j}=\left\{\begin{array}{ll} r_{j} & \quad \text{if episode terminates at step } j+1, \\ r_{j}+\gamma {\max}_{a^{\prime}} \hat{Q}({s_{j}}^{\prime},a^{\prime},{\theta}^{-}) & \quad \text{otherwise}, \end{array} \right. $$

3b $$ \text{loss} = \left( y_{j}-Q(s_{j},a_{j},\theta)\right)^{2}. $$

The minimization of the loss function is performed using gradient descent. 
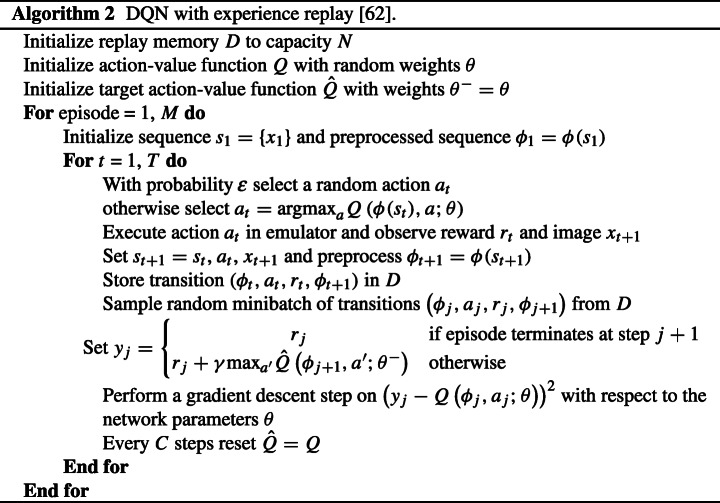


The implemented RL agent shown in Fig. 10 has two main modules: a feature extraction module and action decoder module. The feature extraction module consists of a series of fully-connected layers (dense layers). To improve the stability of training and speed of convergence, each fully-connected layer is followed by a batch normalization layer. This layer lets the network decide the best normalization strategy for the output of the previous layer before going through a ReLU non-linearity.

### Simulation-based validation

The deep network was implemented using PyTorch 0.4 framework under Python 3.6 and trained using NVIDIA GTX1070TI graphics card for one million episodes. A four-layer neural network was used with [128, 128, 64, 32] neurons per each layer, respectively. A batch size of 4096 state-action-reward-next state quadruples, a learning rate of 7e-4, an experience replay memory of one million quadruple, and a discount factor *γ* of 0.999 were used. An *𝜖*-greedy policy was applied and starts with a high *𝜖* of 0.9 to encourage state exploration. Then, it decays exponentially as the number of training iterations increases until it reaches 0.005, which exploits the best action taken by RL agent.

Before testing the model on the actual school students, it was trained using a validated student simulator. The action of the RL agent and the output of Algorithm 1 are passed to the simulator at the beginning of each episode. As the simulator outputs, the state, i.e, interactions measurements analogous to those expected by real students, are produced. A corresponding reward value is also produced to aid the convergence of the RL algorithm. Both the simulated state and reward are set based on previous experience of the teachers with students in classroom and non-adaptive e-learning. Initial learning style and initial level (E, M or H) per taxonomy are considered ground truths about the student once generated. While these parameters are randomly generated in the training phase using the simulator, they are determined from the VARK questionnaire and pre-test for real students. The rest of the configuration parameters include thresholds for the components of the student state definition (time, scores, hints, attempts) and level development (academic level improvement, i.e., scores improvement in addition to time, hints and attempts savings) corresponding to the reward as explained in Section 4.2.1.

Each episode starts with a simulated student selected randomly. Then, it is terminated in two ways: either by reaching the final state in which the student masters the lesson and is ready to unlock the next lesson or the depth of unrolling the episodic tree has reached one hundred states (iterations). The decision time step is the completion of at least one exercise for each of the remaining taxonomies, since the student may have completed the advanced level (hard) exercise in some of the taxonomies. Every step or iteration, a new state-action-reward triplet takes place.

The DQN convergence was validated, where the reward is maximized and the episode is faster for high performing students. The algorithm was validated to act correctly for several scenarios, where the student starts from an entry level corresponding to his/her pre-test score and is downgraded/upgraded based on the obtained penalized grades matching his/her initial ground truth level.

## Pilot experiment and experimental work

This section summarizes some state-of-the-art metrics to evaluate learning effectiveness and their results for the two experimental groups (VARK and gamification) and provides answers to the research questions of Section 1. COVID-19 pandemic and social distancing procedures did not allow comparing against control group (traditional classroom teaching only). The number of students who completed the whole experiment is 26 students in grade 3, whose age ranges from 9-10 years old . Although the sample size is small, some of the reviewed studies reported their results on case studies of the same range [21, 22, 36, 57]. The students were given IDs from 1 to 13 in VARK group and 14 to 26 in gamification group, where the subdivision among the two groups is random. The experiment is carried out for two units covering 8 lessons over the course of around 2 months. Unit 1 is entitled Multiplication and Division with the lessons: Multiplying by 10, Multiplying by 100, Multiplying by 1000, Multiplying a 2- digit number or more by a 1-digit number, Even numbers and odd numbers, and Dividing a number by a 1-digit number. Unit 2 is entitled Geometry with the lessons: The perimeter and The area.

### Aggregated-level academic performance and learning effectiveness indicators

In this subsection, effectiveness is assessed through the score, as performance level indicator, time spent, as effort level indicator, and both, as learning efficiency indicator, in pre-and post-tests, which are summed for both units.

#### Pre- and post-test scores

**Fig. 11:**
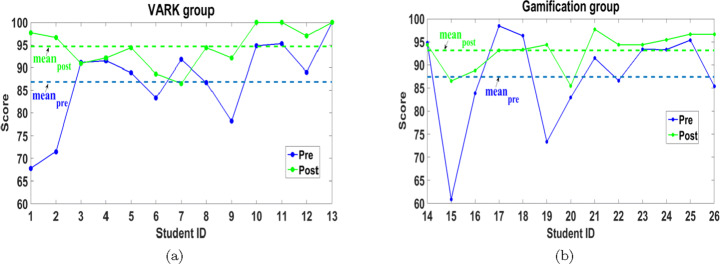
Pre and post test scores of (a) VARK and (b) gamification groups

Figure 11 shows that most students get higher post-test scores than the pre-test scores for both groups. Consequently, the mean of post-test scores is higher than the mean of the pre-test scores by 8.92% and 6.61% for the VARK and gamification groups, respectively.

#### Normalized learning gain (*NLG*)

**Fig. 12:**
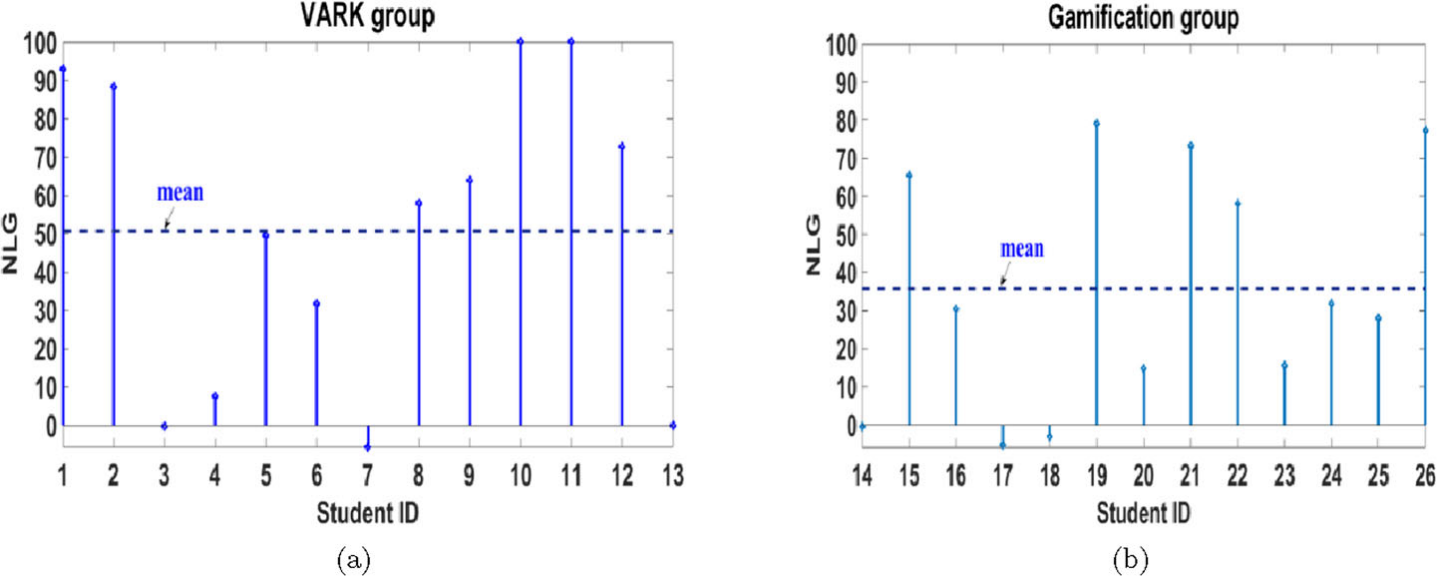
NLG of (a) VARK and (b) gamification groups

*NLG* [53] is given by:

4 $$ {\textit{NLG}}=\left\{ \begin{array}{ll} \frac{{Post}-{Pre}}{100-{Pre}} \times 100, \quad & \qquad {\textit{Post}} \geq {Pre}, \\ \frac{{Post}-{Pre}}{{Pre}} \times 100, \quad & \qquad {\textit{Post}} < {Pre} \end{array} \right., $$

where *Pre* and *Post* refer to the students’ test scores before and after APPEAL’s experiment, respectively; and 100 is the maximum score. *NLG* is averaged for the two units and plotted (in percent) for each student individually in a discrete plot as shown in Fig. 12. *NLG*= 100 means full mark in the post-test. *NLG*= 0 when both scores are equal, i.e., no enhancement in the score. It is negative when the score deteriorates. Figure 12 shows that the majority of the students achieve a high *NLG*; 61.54% of VARK group students achieved *NLG* higher than 50% and 38.46% of gamification group students achieved *NLG* higher than 35%. It can be inferred from Fig. 12 that, using *NLG*, the improvement in the scores of VARK group exceeds gamification group.

#### Pre- and post-test completion time

The test scores are not always enough to assess learning effectiveness. For example, a student may get the same score but finalizes the test in shorter time, which can be considered as an increase in learning efficiency. In other cases, students may not be careful enough in solving the post-test and finish it quickly with mistakes due to boredom or having no obligation on getting high grades. They might be only caring about finishing the experiment more than getting high grades as it is a voluntary experiment not related to the official grading system due to the exceptional circumstances of COVID-19. To overcome these obstacles, time should be considered as well.

**Fig. 13:**
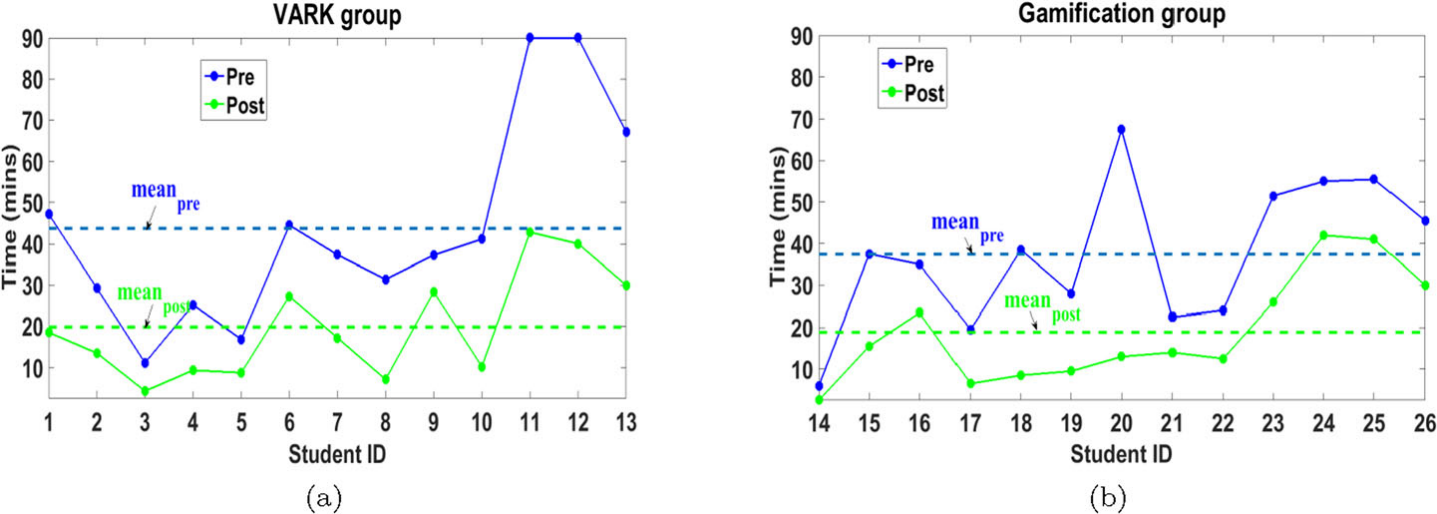
Pre- and post-tests completion time of (a) VARK and (b) gamification groups

First, we consider the completion time of both pre and post tests for both groups in Fig. 13, which indicates that 100% of the students spent shorter time in the post-test than the pre-test for both VARK and gamification groups. Consequently, the mean of post-test times is lower than the mean of the pre-test times by 54.69% and 49.68% in VARK and gamification groups, respectively. In addition, the mean of the post-test time is roughly equal for both groups, which can be considered an indication of consistency in the test completion time after APPEAL experiment.

#### Learning efficiency (LE)

**Fig. 14:**
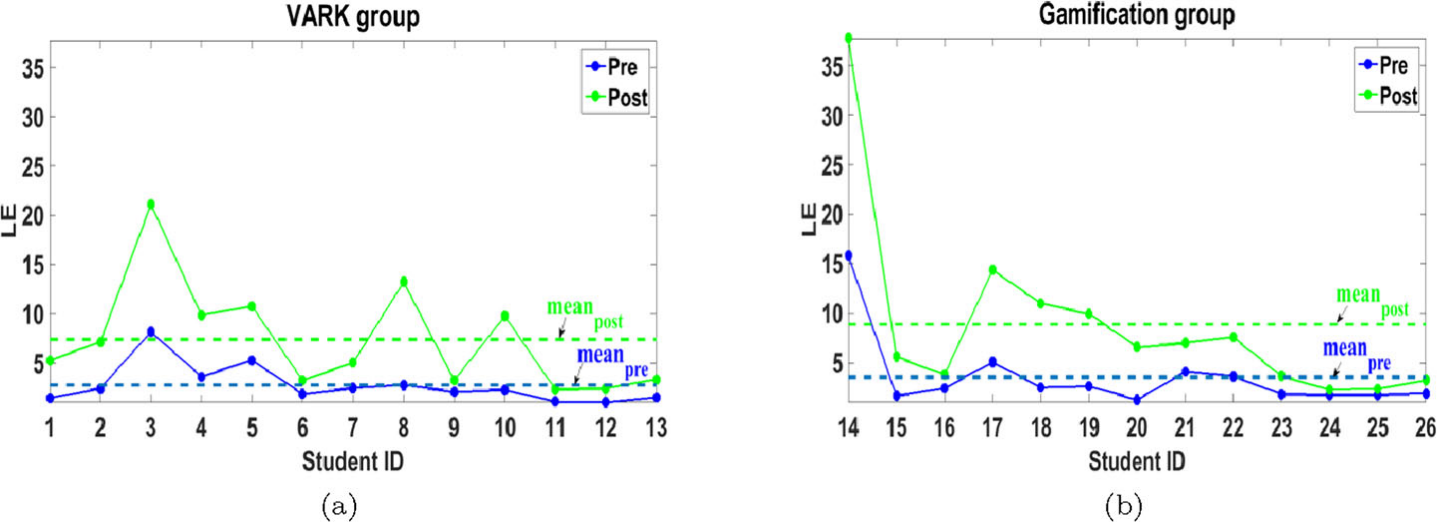
Pre and post LE of (a) VARK and (b) gamification groups

As a collective metric, a definition of LE, which includes the time spent in solving the pre and post-tests as well as the score, is also investigated. In this work and inspired from [24, 53], we define LE as Score/Time, which can be more indicative in the previously mentioned cases. Figure 14 shows the improvement of LE values by 168.59% and 149.72% for VARK and gamification groups, respectively.

These findings provide an answer to RQ1 for both groups, where APPEAL helps improving aggregated-level academic performance and learning effectiveness indicators (pre- and post-test scores, completion time and learning efficiency). The enhancement in scores, time, and accordingly LE for both groups can be attributed to a combination of several insights:

- APPEAL experiment improves student performance in both score and time.
- Scores in pre-test were already quite high, which indicates a high percentage of the students with advanced levels; yet, they spent longer time to solve the test. Hence, there is a higher margin and chance of enhancement in time than scores resulting in better LE.
- Small sample size.
- Similar ranges of time savings were obtained in [27] when comparing two systems with similar content, but different navigation strategies.

LE can be similarly explained as it is a dependent parameter. Although the mean of the LE in the post-test for gamification group is higher than VARK, the relative improvement in the mean of LE of VARK group exceeds gamification group due to having similar relation between pre-test LE means (gamification pre-test LE mean higher than VARK). Moreover, the mean *NLG* equals 50.64% and 35.74% for VARK and gamification groups, respectively. So, in conclusion, although APPEAL experiment helps both groups improving their academic level using different learning effectiveness measures, VARK group exhibits more improvement compared to gamification group.

### Statistical analysis and data dispersion for academic performance and learning effectiveness indicators

The relatively small sample size represents a challenge against performing statistical analysis and quantifying data dispersion or variability. To further analyze the test scores statistically, box-and-whisker plot is used [46], which is more suitable than other explanatory data analysis tools for asymmetrical or highly skewed distributions with possible outliers. The spacings between the different parts of the box indicate the degree of dispersion (spread) and skewness in the data.

**Fig. 15:**
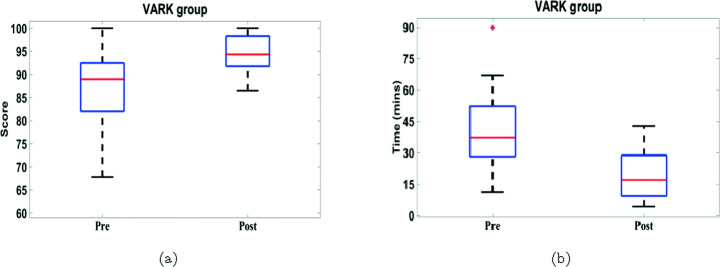
Statistics of pre-and post-test (a) score and (b) time for VARK group

By comparing pre and post-tests of the VARK group, Fig. 15 indicates that APPEAL experiment helps increase the median, minimum and maximum bounds and quartiles of the student scores. In addition, APPEAL experiment reduces the students’ scores dispersion as they become more clustered around the median especially for the lower half of the scores below the median. Similarly, the median, minimum and maximum bounds and quartiles as well as the dispersion of students’ completion times decreased in the post-test compared to pre-test. Table 2 further indicates the positive impact of APPEAL experiment on score and completion time of VARK group, where the medians of scores and completion time improve by 6.06% and 53.89%, respectively. The variation between minimum and maximum (range) reduced with improvement of 58.15% and 31.18% for score and completion time, respectively. Scores and completion time in post-test are more clustered around the median, i.e., Interquartile range (IQR) reduced, than pre-test with improvement of 38.26% and 18.01%, respectively.

**Table 2:** Boxplot summary and relative improvement of pre- and post-test score and time statistics for VARK group

Metric	Median			Range			IQR		
	Pre	Post	Relative Improvement (%)	Pre	Post	Relative Improvement (%)	Pre	Post	Relative Improvement (%)
Score	89	94.39	**6.06**	32.22	13.485	**58.15**	10.4888	6.4762	**38.26**
Time	37.315	17.2065	**53.89**	55.93	38.49	**31.18**	23.8963	19.5915	**18.01**

**Fig. 16:**
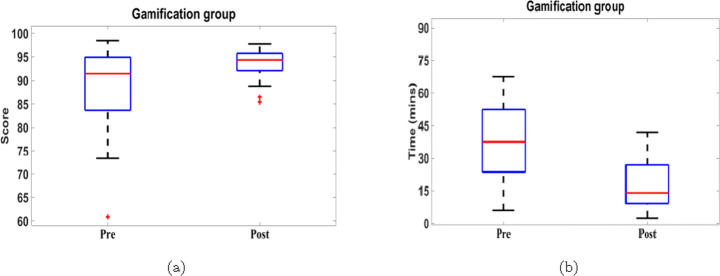
Statistics of pre-and post-test (a) score and (b) time for gamification group

Similar level of improvement in the statistical properties is noticed while studying the impact of the APPEAL experiment on the gamification group. Figure 16 and Table 3 can be described similarly achieving an improvement of 3.18% and 62.67% in the medians of score and completion time, respectively. The range reduced with improvement of 64.43% and 35.77% for score and completion time, respectively. The IQR reduced with improvement of 67.47% and 38.26%, respectively.

**Table 3:** Boxplot summary and relative improvement of pre- and post-test score and time statistics for gamification group

Metric	Median			Range			IQR		
	Pre	Post	Relative Improvement (%)	Pre	Post	Relative Improvement (%)	Pre	Post	Relative Improvement (%)
Score	91.485	94.39	**3.18**	25.12	8.935	**64.43**	11.2962	3.6750	**67.47**
Time	37.5	14	**62.67**	61.5	39.5	**35.77**	28.75	17.75	**38.26**

These findings provide an answer to RQ2 for both groups, where APPEAL helps improving the data dispersion for the academic performance and learning effectiveness in terms of score and time.

### Disaggregated-level academic performance and learning effectiveness indicators

It was found that learners with low prior knowledge can benefit much more than those with high prior knowledge [54, 64]. Around 60% and 76.93% of the students in VARK and gamification groups, respectively, got high scores in the pre-test of both units and, hence, started at the advanced level or hard exercises already. These advanced level students preserve their level. Table 4 gives a list of the students who got pre-test scores less than 75%, which represent 30.77% from the total number of students in VARK group and 23.08% from gamification group. It can be inferred from Table 4 that the post-test level of 100% of these students improves than their pre-test level. In addition, 100% of the students reached the advanced level and became capable of solving hard exercises correctly. Table 5 presents examples extracted from the exercises log data showing how these students’ performance gradually improves in different taxonomies and lessons. These are the real scenarios corresponding to the hypothetical scenarios of Section 4.3. These findings provide an answer to RQ3, where APPEAL helps improving disaggregated-level academic performance and learning effectiveness indicators for each student on lesson and exercises level.

**Table 4:** Students level improvement summary

ID	Group	Unit 1		Unit 2	
		Pre-test level	Post-test level	Pre-test level	Post-test level
1	VARK	-	-	E	H
2	VARK	-	-	E	H
6	VARK	-	-	M	H
9	VARK	-	-	M	H
15	Gamification	M	H	M	H
19	Gamification	-	-	E	H
20	Gamification	-	-	M	H

**Table 5:**
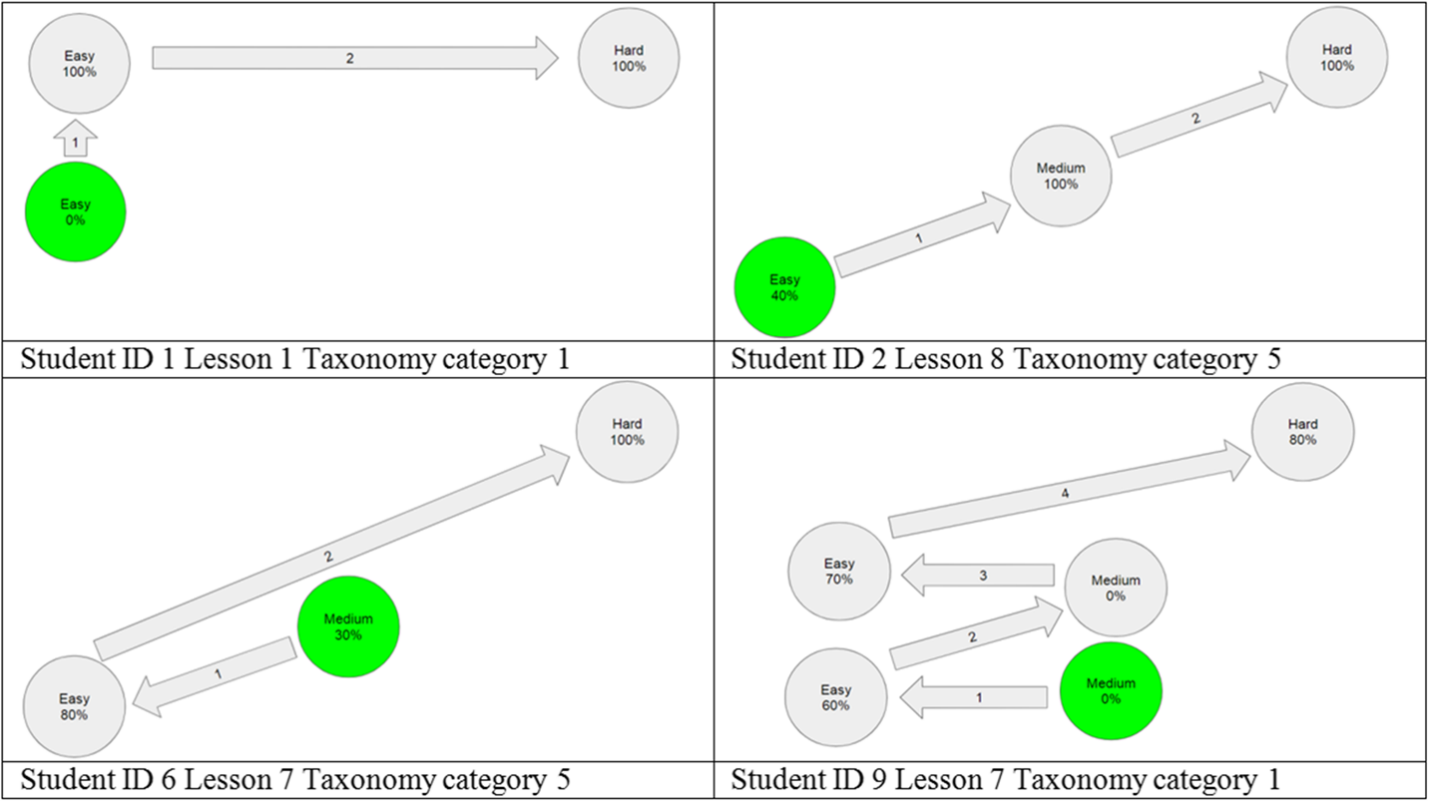
Performance improvement examples from exercises log data

### Student engagement and satisfaction indicators

APPEAL survey tests the satisfaction level among the students and assesses the different hypotheses of the project from their perspective. It consists of 11 questions, which have graduated choices on LIKERT scale. Table 6 shows the students’ responses to the different hypotheses. The green color corresponds to strongly agree and the red color corresponds to strongly disagree. It can be inferred that there is 95% and 85.9375% overall satisfaction about APPEAL in VARK and gamification groups, respectively. Table 7 provides a detailed comparison, where 94% of the VARK group and 75% of the gamification group were satisfied by the presentation style and engagement. 95% of the VARK group and 87.5% of the gamification group were satisfied by the exercises scaffolding, navigation and feedback. Combining these two dimensions results in 94% VARK satisfaction compared to 75% gamification satisfaction. The less satisfied percentage of students mostly selected “neutral” and only around 6.25% of the gamification group were unsatisfied by presentation style and engagement and 2.5% were unsatisfied by exercises scaffolding, navigation and feedback. For VARK group, only 4% were unsatisfied by exercises scaffolding, navigation and feedback.

**Table 6:**
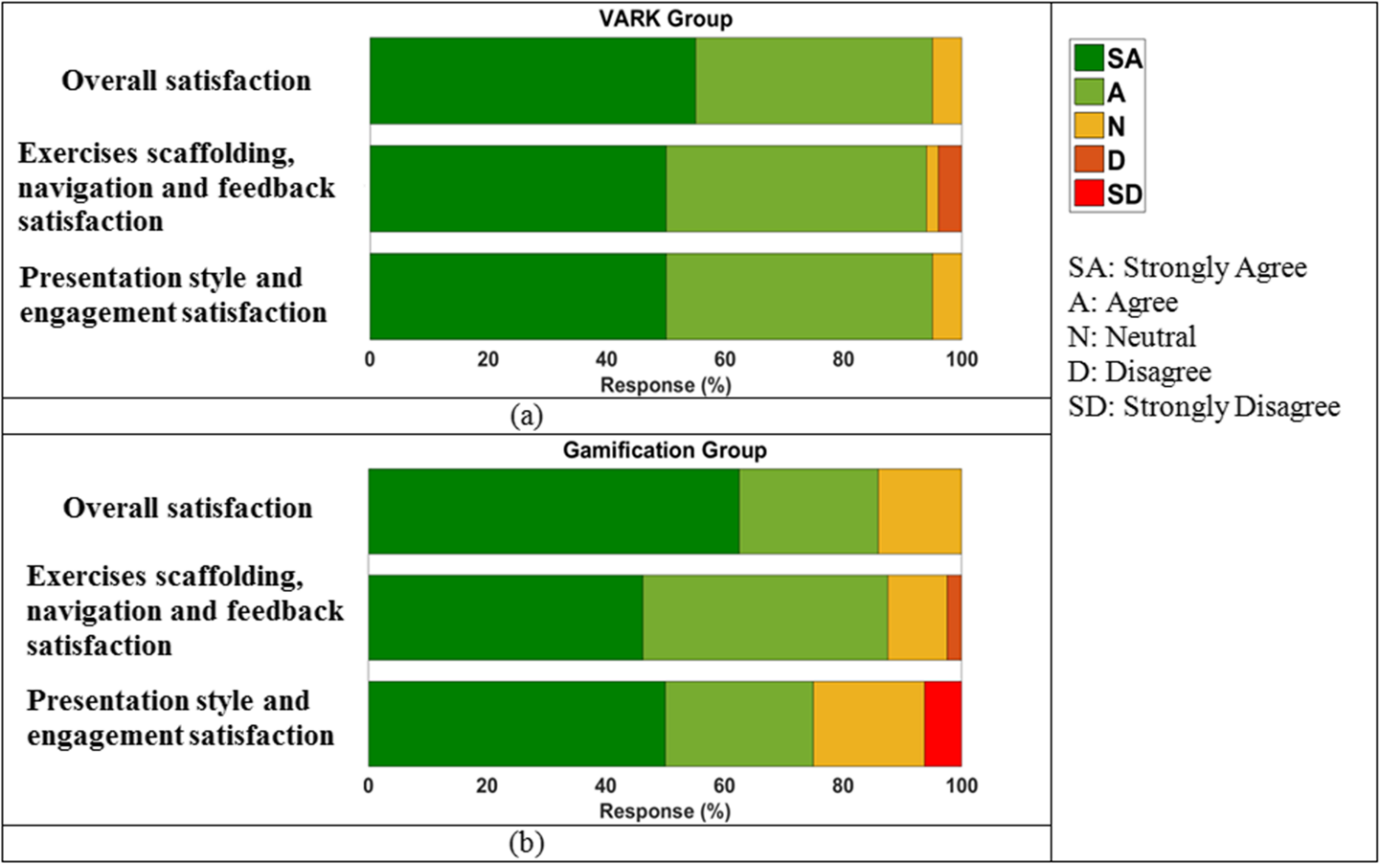
Satisfaction survey results for (a) VARK and (b) Gamification groups

**Table 7:** Comparison of the satisfaction level for both groups

Point of view	VARK	Gamification
Overall satisfaction	95%	85.9375%
Presentation style and engagement satisfaction	94%	75%
Exercises scaffolding, navigation and feedback satisfaction	95%	87.5%
Both presentation and navigation	94%	75%

These findings provide an answer to RQ4, where APPEAL achieves good student engagement and satisfaction indicators. At first, it was expected that gamification for third grade students may yield better results by increasing engagement and satisfaction. However, the learning effectiveness measures of Section 5.1 and satisfaction survey results come in favor of VARK. This may be attributed to the initial learning styles of gamification group, which reports more than 30% for Read/Write learning style, while gamified content requires more interaction and kinesthetic abilities.

The conducted experiment comes in favor of offering different/mixed presentation modalities according to AI-based update of learning style and dynamic preferences than offering gamified content. From the relative improvement in test score, completion time, *NLG* and LE and satisfaction level, it can be inferred that VARK approach is slightly more suitable to achieve higher learning effectiveness compared to gamification. For both groups, the adaptive personalized exercises navigation played an important role in achieving this relative improvement and high satisfaction.

## Conclusions

This paper proposed an AI-based Adaptive Personalized Platform for an Effective and Advanced Learning (APPEAL) for school students, which achieves more effective learning with reduced time spent, improved grades and satisfaction level. APPEAL is built as a dedicated website based on Moodle including a front-end interface, back-end algorithms and API. APPEAL provides user accounts with different roles (student and teacher). A dynamic learner model was built including cognitive (learning style); behavior (effort, support and performance) and affective (engagement) categories of the student personal traits. APPEAL presents a new combination of the following:

- VARK adaptive personalized content presentation on one hand or gamification on the other hand.
- Adaptive personalized content presentation employs a DQN RL AI implementation.
- Adaptive personalized exercises difficulty scaffolding and navigation (through skipping/hiding less difficult exercises) and adaptive feedback (through hints and messages), as well as reattempting exercises till mastery employing an online rule-based decision making based on student interactions.

The performance of APPEAL was experimentally tested in regard to the four research questions and proved to achieve an improvement in learning effectiveness in a real life pilot experiment:

- For RQ1, data analysis demonstrated that APPEAL experiment has a positive impact on the performance of all participants in the post-test compared to the pre-test. An improvement in the mean of test scores (9% VARK and 7% gamification), completion time (55% VARK and 50% gamification) and LE (169% VARK and 150% gamification) were reported. In addition, mean *NLG* of 51% and 36% were reported for VARK and gamification groups, respectively.
- As for RQ2, around 31% from the total number of students in the VARK group and 23% from the gamification group started the experiment with easy and medium levels in the pre-test. APPEAL helped 100% of these students to enhance their performance and reach hard (advanced) in the post-test.
- Statistically, APPEAL experiment improves the median and reduces the dispersion, which provides an answer to RQ3.
- Regarding RQ4, 95% and 86% satisfaction level were reported for VARK and gamification groups, respectively.
- Irrespective of the initial learning style from the questionnaire, APPEAL experiment, with the adaptive personalized RL algorithm that allows personalized choices of presentation styles, helped the students improve their academic performance to higher levels.
- Both VARK and gamification groups witnessed academic and satisfaction level improvement after APPEAL experiment.
- More improvement was reported in adaptive personalized VARK presentation group, where interactive activities and games in the kinesthetic presentation can also provide engagement, while keeping other presentation styles available, when needed.

The obtained results suggest an improvement in the post-test score (academic performance), time (effort) and hence efficiency compared to the pre-test, promising *NLG* values, statistics and satisfaction level for both groups, especially VARK. However, these results should be cautiously taken as the sample size was not as big as planned due to the impact of COVID-19 pandemic and social distancing procedures on schools. The number of students who voluntarily started and completed the experiment was 26 students, coordinating with their school. Yet, the motivation factor of relating their performance to a typical grading system was absent. Given the duration of this pilot experiment (2 months), the results seem reasonable, carry a good potential and encourage a future extension.

The different presentation styles offered by APPEAL can support students with physical and cognitive disabilities such as aural or visual processing deficits. In addition, the following items can be considered for future work:

- The data collected from real students can be used to enhance the training of the model and implement another supervised model as a benchmark for comparison.
- An experiment involving a control group with conventional classroom teaching against experimental groups, with larger number of participants, can be set up given previous history about the students who will take part in the experiment. We anticipate that the results reflect similar improvement, yet, with more variations among different students’ levels.
- Dual or compound action RL for both presentation and exercises navigation can be addressed.
- Deeper aspects of personalized gamification [31] can be investigated and combined in the AI implementation aspiring for better results for gamification group.
- Multi-task DQN RL can be applied to predict both VARK and difficulty optimal actions.
- Multi-agent RL can be applied in Computer-Supported Collaborative Learning (CSCL) for group learning purposes.
- APPEAL can be extended to other age groups.

## Appendix: List of symbols

**Table:**

T1-T6	Bloom’s taxonomy with 6 categories
E-M-H	Levels of exercise difficulty: Easy-Medium-Hard
*tax*	Taxonomy category
*P**r**e*	Pre-test score of each unit
*Post*	Post-test score of each unit
*d**i**f**f**i**c**u**l**t**y*	Difficulty level of the exercise that appear to the student at entry level per *tax*
*d**i**f**f**i**c**u**l**t**y*_*P**r**e*_	Initial value of *d**i**f**f**i**c**u**l**t**y*
*g**r**a**d**e*	Student grade in the exercise in the interval [0, 1]
*g**r**a**d**e*_*p**e**n**a**l**i**z**e**d*_	Penalized grade that implicitly includes the attempts and hints
*h**i**n**t**s*	Number of hints taken by the student up to the available 4 attempts
*t**h*_*g*1_ (*t**h*_*g*2_)	Threshold grades corresponding to upgrading, no change, or downgrading *d**i**f**f**i**c**u**l**t**y*
*NLG*	Normalized Learning Gain
LE	Learning Efficiency
< *S*, *A*, *R*, *T*, *γ* >	MDP tuple (states, actions, reward, transition probabilities, discount factor)
$P(s^{\prime }|s,a)$	Probability distribution over next states given current state and action
*Q*(*s*, *a*)	Action value function
*𝜃*	Parameters/weights of the online network
*Q*(*s*,⋅; *𝜃*)	Action value function for any set of actions output by the function approximation parameterized by *𝜃*
$\hat {Q}(s,\cdot ;{\theta }^{-})$	Action value function for any set of actions output by the target network parameterized by *𝜃*
*𝜃*^−^	Parameters/weights of target network
*C*	Number of steps after which the target network is copied from the online network
*D*	Replay buffer
*N*	Capacity of replay buffer
*M*	Number of episodes
*𝜖*	Probability of random action selection
*π*	RL policy
$<s,a,r,s^{\prime }>$	Experience tuple (state-action-reward-next state)

## References

[1] Alhathli M, Masthoff J, Siddharthan A (2017) Should learning material’s selection be adapted to learning style and personality?. In: Adjunct publication of the 25th conference on user modeling, adaptation and personalization, pp 275–280

[2] Analytica O (2016) Gamification and the future of education. World Government Summit

[3] Arqub OA, Abo-Hammour Z (2014). Numerical solution of systems of second-order boundary value problems using continuous genetic algorithm. Inf Sci.

[4] Arqub OA, Al-Smadi M (2020) Fuzzy conformable fractional differential equations: novel extended approach and new numerical solutions. Soft Computing

[5] Balasubramanian V, Anouncia SM (2016) Learning style detection based on cognitive skills to support adaptive learning environment–a reinforcement approach. Ain Shams Engineering Journal

[6] Beck J, Woolf BP, Beal CR (2000). Advisor: a machine learning architecture for intelligent tutor construction. AAAI/IAAI.

[7] Bimba AT, Idris N, Al-Hunaiyyan A, Mahmud RB, Shuib NLBM (2017). Adaptive feedback in computer-based learning environments: a review. Adapt Behav.

[8] Bloom BS (1968). Learning for mastery. instruction and curriculum. regional education laboratory for the carolinas and virginia, topical papers and reprints, number 1. Evaluation comment.

[9] Bloom BS (1984). The 2 sigma problem: The search for methods of group instruction as effective as one-to-one tutoring. Educ Res.

[10] Bocconi S, Kampylis P, Punie Y (2013). Framing ict-enabled innovation for learning: the case of one-to-one learning initiatives in europe. Eur J Educ.

[11] Chen CH (2014) An adaptive scaffolding e-learning system for middle school students? physics learning. Australasian Journal of Educational Technology 30(3)

[12] Chu YS, Yang HC, Tseng SS, Yang CC (2014). Implementation of a model-tracing-based learning diagnosis system to promote elementary students’ learning in mathematics. J Educ Technol Soc.

[13] Covaci A, Ghinea G, Lin CH, Huang SH, Shih JL (2018). Multisensory games-based learning-lessons learnt from olfactory enhancement of a digital board game. Multimed Tools Appl.

[14] Dolenc K, Aberšek B (2015). Tech8 intelligent and adaptive e-learning system: Integration into technology and science classrooms in lower secondary schools. Comput Educ.

[15] Early DM, Maxwell KL, Burchinal M, Alva S, Bender RH, Bryant D, Cai K, Clifford RM, Ebanks C, Griffin JA (2007). Teachers’ education, classroom quality, and young children’s academic skills: Results from seven studies of preschool programs. Child Dev.

[16] El-Tantawy S, Abdulhai B, Abdelgawad H (2014). Design of reinforcement learning parameters for seamless application of adaptive traffic signal control. J Intell Transp Syst.

[17] Faiella F, Ricciardi M (2015) Gamification and learning: a review of issues and research. Journal of e-Learning and Knowledge Societ 11(3)

[18] Feng M, Cui W, Wang S (2018) Adaptive learning goes to China. In: International Conference on Artificial Intelligence in Education. Springer, pp 89–93

[19] Fößl T, Ebner M, Schön S, Holzinger A (2016). A field study of a video supported seamless-learning-setting with elementary learners. J Educ Technol Soc.

[20] Gylfason T (2001). Natural resources, education, and economic development. Eur Econ Rev.

[21] Hsieh SW, Jang YR, Hwang GJ, Chen NS (2011). Effects of teaching and learning styles on students? reflection levels for ubiquitous learning. Comput Educ.

[22] Hsieh YH, Yi-Chun L, Hou HT (2015). Exploring elementary-school students’ engagement patterns in a game-based learning environment. J Educ Technol Soc.

[23] Huang CJ, Liu MC, Chang KE, Sung YT, Huang TH, Chen CH, Shen HY, Huang KL, Liao JJ, Hu KW (2010). A learning assistance tool for enhancing ict literacy of elementary school students. J Educ Technol Soc.

[24] Hubalovsky S, Hubalovska M, Musilek M (2019). Assessment of the influence of adaptive e-learning on learning effectiveness of primary school pupils. Comput Hum Behav.

[25] Huey-Min W, Bor-Chen K, Su-Chen W (2017). Computerized dynamic adaptive tests with immediately individualized feedback for primary school mathematics learning. J Educ Technol Soc.

[26] Iglesias A, Martínez P, Aler R, Fernández F (2009). Learning teaching strategies in an adaptive and intelligent educational system through reinforcement learning. Appl Intell.

[27] Iglesias A, Martínez P, Aler R, Fernández F (2009). Reinforcement learning of pedagogical policies in adaptive and intelligent educational systems. Knowl Based Syst.

[28] James WB, Blank WE (1993). Review and critique of available learning-style instruments for adults. New Directions for Adult and Continuing Education.

[29] Johnson L, Becker SA, Estrada V, Freeman A (2014) NMC horizon report: 2014 K. The New Media Consortium

[30] Keefe JW (1989). Learning style profile handbook: II.

[31] Knutas A, Van Roy R, Hynninen T, Granato M, Kasurinen J, Ikonen J (2019). A process for designing algorithm-based personalized gamification. Multimed Tools Appl.

[32] Koć-Januchta MM, Höffler TN, Eckhardt M, Leutner D (2019). Does modality play a role? visual-verbal cognitive style and multimedia learning. J Comput Assist Learn.

[33] Krathwohl DR, Anderson L W (2009) A taxonomy for learning, teaching, and assessing: A revision of Bloom’s taxonomy of educational objectives. Longman

[34] Kulik JA, Fletcher J (2016). Effectiveness of intelligent tutoring systems: a meta-analytic review. Rev Educ Res.

[35] Leite WL, Svinicki M, Shi Y (2010). Attempted validation of the scores of the vark: Learning styles inventory with multitrait–multimethod confirmatory factor analysis models. Educ Psychol Meas.

[36] Lin HT, Lee PM, Hsiao TC (2015) Online pedagogical tutorial tactics optimization using genetic-based reinforcement learning. The Scientific World Journal 2015 10.1155/2015/352895 PMC4439506 26065018

[37] Lin CH, Liu EZF, Chen YL, Liou PY, Chang M, Wu CH, Yuan SM (2013). Game-based remedial instruction in mastery learning for upper-primary school students. J Educ Technol Soc.

[38] Mnih V, Kavukcuoglu K, Silver D, Rusu AA, Veness J, Bellemare MG, Graves A, Riedmiller M, Fidjeland AK, Ostrovski G (2015). Human-level control through deep reinforcement learning. Nature.

[39] Mosharraf M (2016). Tuning primary learning style for children with secondary behavioral patterns. Interdisciplinary Journal of e-Skills and Lifelong Learning.

[40] Neumann MM (2018). Using tablets and apps to enhance emergent literacy skills in young children. Early Child Res Q.

[41] Nolan J, McBride M (2014). Beyond gamification: reconceptualizing game-based learning in early childhood environments. Inf Commun Soc.

[42] Normadhi NBA, Shuib L, Nasir HNM, Bimba A, Idris N, Balakrishnan V (2019). Identification of personal traits in adaptive learning environment: Systematic literature review. Comput Educ.

[43] Okpo JA, Masthoff J, Dennis M, Beacham N (2018). Adapting exercise selection to performance, effort and self-esteem. New Rev Hypermedia Multimed.

[44] Pan WF (2017). The effects of using the kinect motion-sensing interactive system to enhance english learning for elementary students. J Educ Technol Soc.

[45] Papoušek J, Pelánek R (2017) Should we give learners control over item difficulty?. In: Adjunct Publication of the 25th Conference on User Modeling, Adaptation and Personalization, pp 299–303

[46] Potter K, Hagen H, Kerren A, Dannenmann P (2006). Methods for presenting statistical information: The box plot. Visualization of large and unstructured data sets.

[47] Prabha SL, Shanavas AM (2014). Educational data mining applications. Operations Research and Applications: An International Journal (ORAJ).

[48] Premlatha K, Geetha T (2015). Learning content design and learner adaptation for adaptive e-learning environment: a survey. Artif Intell Rev.

[49] Pruet P, Ang CS, Farzin D (2016). Understanding tablet computer usage among primary school students in underdeveloped areas: Students’ technology experience, learning styles and attitudes. Comput Hum Behav.

[50] Sarwar S, Qayyum ZU, García-castro R, Safyan M, Munir RF (2019). Ontology based e-learning framework: A personalized, adaptive and context aware model. Multimed Tools Appl.

[51] Shawky D, Badawi A (2018) A reinforcement learning-based adaptive learning system. In: International Conference on Advanced Machine Learning Technologies and Applications. Springer, pp 221–231

[52] Shawky D, Badawi A (2019) Towards a personalized learning experience using reinforcement learning. In: Machine learning paradigms: Theory and Application. Springer, pp 169–187

[53] Shen S, Ausin MS, Mostafavi B, Chi M (2018) Improving learning & reducing time: A constrained action-based reinforcement learning approach. In: Proceedings of the 26th Conference on User Modeling, Adaptation and Personalization, pp 43–51

[54] Shyr WJ, Chen CH (2018). Designing a technology-enhanced flipped learning system to facilitate students’ self-regulation and performance. J Comput Assist Learn.

[55] Sutton RS (2018). Barto, A G.

[56] Tashtoush YM, Al-Soud M, Fraihat M, Al-Sarayrah W, Alsmirat MA (2017) Adaptive e-learning web-based english tutor using data mining techniques and jackson’s learning styles. In: 2017 8Th international conference on information and communication systems (ICICS). IEEE, pp 86–91

[57] Tetreault JR, Litman DJ (2006) Using reinforcement learning to build a better model of dialogue state. In: 11th Conference of the european chapter of the association for computational linguistics

[58] Thepsatitporn S, Pichitpornchai C (2016). Visual event-related potential studies supporting the validity of vark learning styles’ visual and read/write learners. Adv Physiol Educ.

[59] Turchi T, Fogli D, Malizia A (2019). Fostering computational thinking through collaborative game-based learning. Multimed Tools Appl.

[60] VARK (2018) http://vark-learn.com/the-vark-questionnaire/the-vark-questionnaire-for-younger-people/. Accessed 30 April 2020

[61] Vandewaetere M, Clarebout G (2014) Advanced technologies for personalized learning, instruction, and performance. In: Handbook of research on educational communications and technology. Springer, pp 425–437

[62] Vandewaetere M, Desmet P, Clarebout G (2011). The contribution of learner characteristics in the development of computer-based adaptive learning environments. Comput Hum Behav.

[63] Wang TH (2014). Developing an assessment-centered e-learning system for improving student learning effectiveness. Comput Educ.

[64] Webb S, Chang ACS (2015). How does prior word knowledge affect vocabulary learning progress in an extensive reading program?. Stud Second Lang Acquis.

[65] Xiao L, Wan X, Lu X, Zhang Y, Wu D (2018). Iot security techniques based on machine learning: How do iot devices use ai to enhance security?. IEEE Signal Proc Mag.

[66] Young SSC, Ku HH (2008). A study of uses of ict in primary education through four winning school cases in the Taiwan schools cyberfair. J Educ Technol Soc.

